# Immuno-epigenetic paradigms in coronavirus infection

**DOI:** 10.3389/fimmu.2025.1596135

**Published:** 2025-09-03

**Authors:** Swati Gupta, Hassan A. Hemeg, Farhat Afrin

**Affiliations:** ^1^ Centre for Interdisciplinary Sciences, JIS Institute of Advanced Studies and Research, JIS University, Santragachi, Howrah, West Bengal, India; ^2^ Department of Medical Laboratory Technology, Faculty of Applied Medical Sciences, Taibah University, Medina, Saudi Arabia

**Keywords:** coronavirus, epigenetics, immune evasion, mucosal immunity, pathogenesis, host-pathogen interactions, epigenetic drugs, COVID-19

## Abstract

Coronavirus Disease 2019 (COVID-19) is caused by the Severe Acute Respiratory Syndrome Coronavirus-2 (SARS-CoV-2), a novel member of the Coronaviridae family. The viral genome encodes both structural proteins, such as spike, membrane, hemagglutinin, and envelope, as well as non-structural proteins that include auxiliary proteins and replicase essential for viral replication. While immunization campaigns have mitigated the spread of the virus, therapeutic interventions remain critical for managing outbreaks and preventing long-term health consequences. Despite extensive global research into the genome, structure, entry process, and replication mechanisms of SARS-CoV-2, key aspects such as the roles of membrane lipids in viral entry, packaging, and release, as well as the metabolic alterations in infected cells, remain poorly understood. Epigenetics, the study of heritable phenotypic changes driven by genetic and non-genetic factors, plays a pivotal role in shaping host responses to SARS-CoV-2 infection. Epigenetic modifications, such as histone methylation and acetylation, DNA and RNA methylation, chromatin remodeling, and non-coding RNA regulation, significantly influence gene expression in infected host cells. These reversible changes orchestrate the host’s antiviral responses and potentially alter susceptibility to COVID-19. This review delves into the immuno-epigenetic modifications occurring in hosts infected with SARS-CoV-2, providing insights into how these changes trigger viral replication and infection processes. By examining the current state of research on the immune-epigenetic landscape of SARS-CoV-2 infections, we highlight the mechanisms by which these modifications affect the host-viral interplay. Furthermore, we propose potential therapeutic targets within the immune-epigenetic pathways that could enhance ongoing efforts to combat COVID-19. Understanding these mechanisms will not only provide a deeper perspective on the virus’s pathogenic strategies but also offer innovative approaches to improve therapeutic interventions. By addressing the gaps in knowledge surrounding immune-epigenetic factors, this review aims to contribute to the development of novel strategies for preventing and managing coronavirus infections and its variants.

## 1 Introduction

The Coronavirus disease 2019 (COVID-19) pandemic, declared a global health emergency in 2019, has profoundly impacted not only the health sector but also economic, social, and cultural aspects of human life worldwide. The unexpected emergence of Severe Acute Respiratory Syndrome Coronavirus-2 (SARS-CoV-2), a novel member of the coronavirus family, impacted every facet of human society globally across medical, business, research and education, games and sports and transportation domains, among others. First identified in Wuhan city of China’s Hubei Province, SARS-CoV-2 is highly sporadic and transmissible, spreading via direct contact or aerosols, and has claimed the lives of approximately 6.3 million people globally (1). The World Health Organization (WHO) declared the outbreak a worldwide health emergency, as cases and fatalities continued to rise at an alarming rate. Early COVID-19 infection and related mortality affected the US, Italy, Spain, and the UK severely; this pattern persisted in the second wave of the pandemic. Conversely, compared to the aforementioned countries, Brazil and India have had higher infection rates with lower death rates (2).

In the two decades preceding 2019, two coronaviruses evolved capable of causing serious respiratory infections in people. SARS-CoV appeared in late 2002 in Guangdong Province, China, causing SARS while another coronavirus, MERS-CoV, which causes Middle East Respiratory Syndrome (MERS), appeared in the Middle East in 2012. The unique epidemiology of SARS-CoV-2 sets it apart from previous coronaviruses, *viz*, SARS-CoV and MERS-CoV (3). While both SARS and MERS caused severe respiratory infections, their outbreaks were more localized and contained, as symptomatic individuals were the primary source of transmission. In contrast, SARS-CoV-2’s ability to spread asymptomatically contributed to its rapid global dissemination, rendering quarantine measures less effective. SARS-CoV-2 is believed to have a zoonotic origin, with bats serving as the likely natural reservoir and transmission occurring through an intermediate species to humans (4). This zoonotic spill over, a major driver of emerging infectious diseases, mirrors the transmission mechanisms of other significant outbreaks, including HIV and influenza. The virus has evolved mechanisms for efficient human-to-human transmission, a key factor behind the scale of the pandemic.

COVID-19 presents with an array of symptoms, including fever with chills, sore throat and cough, running nose, congestion, shortness of breath, altered or loss of taste and smell, headache, muscular or body pain, nausea, and, in severe cases, secondary infections of *Rhizopus* or *Aspergillus*-related mucormycosis (5). Immunocompromised individuals are particularly vulnerable to complications, including COVID-19-associated mucormycosis (CAM). Despite extensive research into the genomic structure, replication mechanisms, and transmission of SARS-CoV-2, several aspects of its pathogenicity, including epigenetic influences, remain poorly understood (6). Epigenetics, involving heritable changes in gene expression without altering the underlying DNA sequence, plays a critical role in host-pathogen interactions.

**Figure 1:**
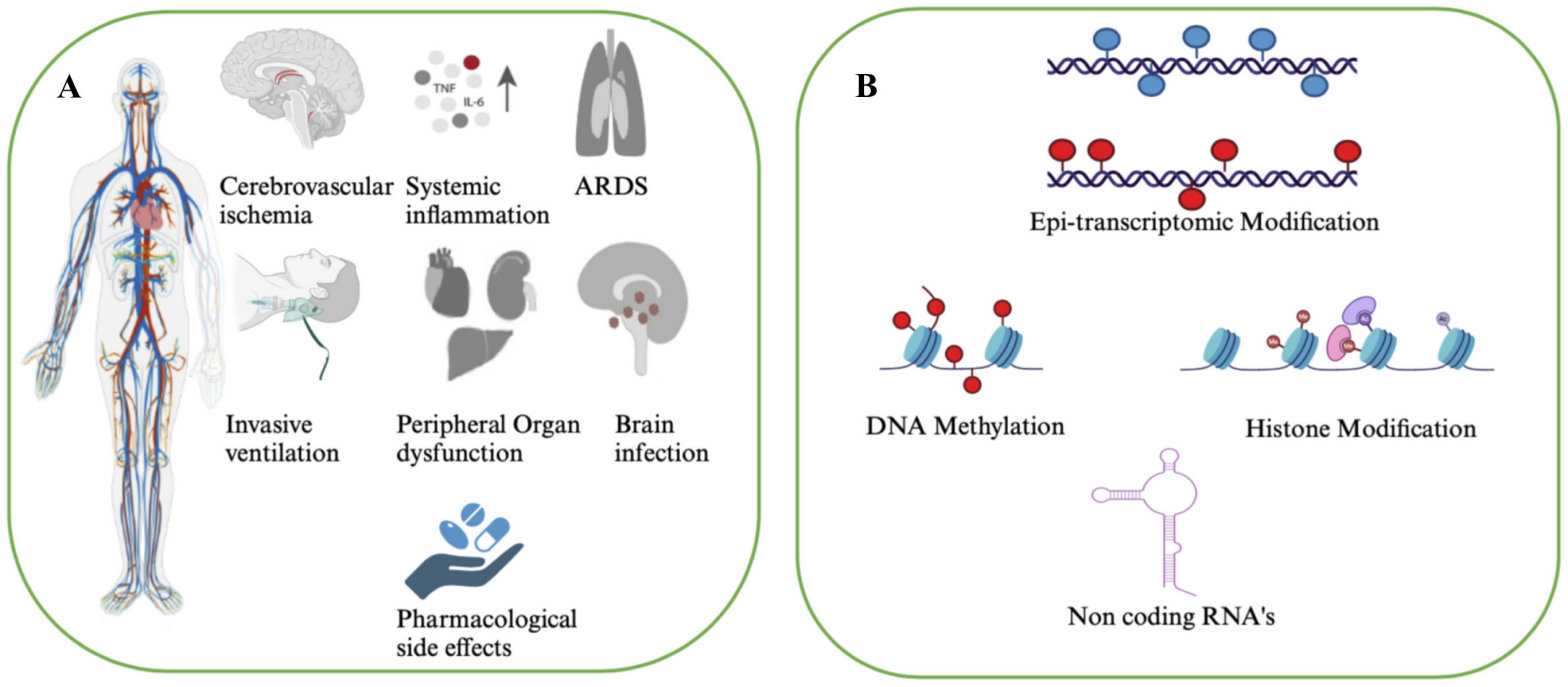
Pathological effects and epigenetic players during SARS-CoV-2 infection. **(A)** By inhibiting the function of angiotensin converting enzyme 2 (ACE2), SARS-CoV-2 may cause the injury of the lung, including pulmonary fibrosis and pulmonary hypertension. Besides, the intestines, heart, kidney, esophagus, bladder, ileum, testis, and adipose tissue may also express ACE2 and may be affected in severe form of COVID-19 in the diseased individuals. **(B)** Aberrant epigenetic signatures that may influence susceptibility to SARS-CoV-2 infection and contribute to disease progression.

Epigenetics encompasses the regulation of gene expression by environmental stimuli such as diet, temperature, humidity, pollution and biological and phenotypic factors like age and sex. This regulation entails the alteration of DNA conformation within chromosomes, allowing or restricting access to transcription factors. Epigenetic modifications, unlike genetic mutations, alter the chromatin structure or the chemical properties of nucleic acids without changing the underlying DNA sequence (7). Key mechanisms of epigenetic regulation include DNA/RNA methylation, histone and epi-transcriptomic modifications, and microRNA (miRNA) expression (**Figure 1**). When an infection occurs, host cells undergo significant changes to counteract or halt viral replication. These responses may include activating innate and adaptive immune mechanisms or, in severe cases, triggering cell death. However, viruses have co-evolved with their hosts, developing autonomous strategies to evade or subvert these antiviral defenses. By reprogramming host cells, viruses create environments conducive to their replication, transforming infected cells into viral factories. While the virus counteracts and manipulates the host cell for its own gain, the host cell strives to contain and contend with the virus. This dynamic tug-of-war leads to epigenetic changes within the host. These alterations regulate the expression of antiviral genes and modulate host cell properties that virus exploits for successful replication and transmission. Moreover, epigenetic changes enable cells to adapt to environmental disruptions by modifying gene expression profiles (8). Thus, most viruses do not directly modify the host’s genetic sequence but instead influence its epigenome, thereby facilitating viral establishment, replication, and persistence. Notably, epigenetic modifications, such as alterations in DNA, histones, and RNA patterns, are reversible and naturally respond to environmental disturbances (9). For example, during infection, the host cell’s epigenome may adapt to re-establish homeostasis.

Infections also induce immune responses that affect metabolic dysregulation besides epigenetic processes. Viral infections often trigger metabolic shifts to enhance cellular energy production, supporting immune responses, while simultaneously inducing epigenetic modifications. These findings highlight the profound metabolic and epigenetic consequences of viral infections, emphasizing the need for further research into epigenetic and metabolic markers (10). Such investigations, particularly involving high-throughput samples and advanced omics technologies, could reveal novel therapeutic targets and improve our understanding of viral pathogenesis and host response. This review explores the specific interplay of the SARS-CoV-2 at the mucosal interface and the immune-epigenetic stratagems employed to evade host immune responses, particularly in the mucosal environment, providing insights into its pathogenesis and potential therapeutic targets.

## 2 Structure of SARS-CoV-2

**Figure 2:**
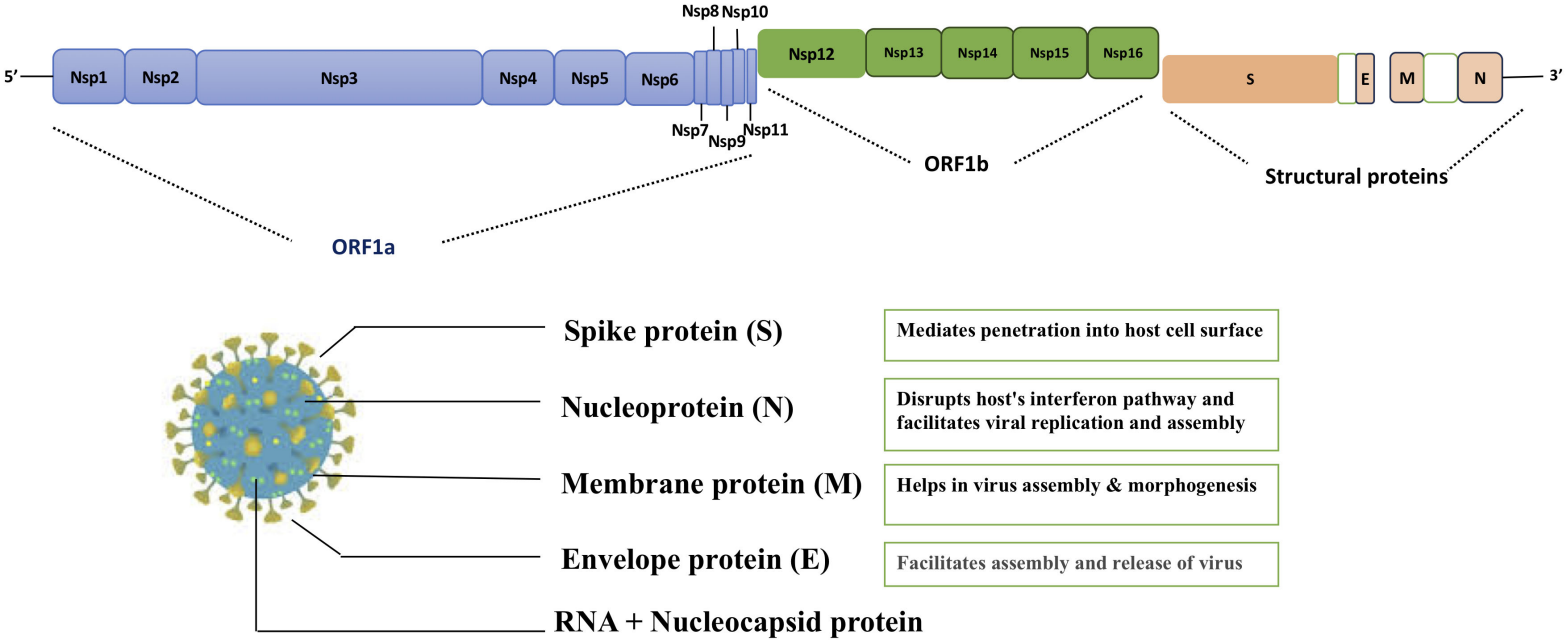
Overview of severe acute respiratory syndrome coronavirus-2 (SARS-CoV-2) structure. 1; Spike protein 2; E, envelope; M, membrane; N, nucleocapsid; RNA, ribonucleic acid; nsps, non-structural proteins; ORF, open-reading frame. The image depicts the key components of the SARS-CoV-2 virus, which causes severe acute respiratory syndrome.

SARS-CoV-2 is a betacoronavirus with a large (~30 kb), single-stranded, positive-sense RNA genome enclosed in a lipid envelope. It shares 96% genome similarity with Bat CoV RaTG13, 79% with SARS-CoV, and 50% with MERS CoV (11, 12). The virus contains four primary structural proteins: nucleocapsid (N), spike (S), membrane (M) and envelope (E). The S, M, and E proteins are embedded in the viral lipid envelope, while the N protein coats the RNA (**Figure 2**). A unique feature of SARS-CoV-2 is the presence of a functional furin cleavage site in the S protein, enhancing its infectivity (13).

### 2.1 Spike protein

The trimeric S glycoprotein (1255 aminoacid {aa} residues) mediates viral entry. Its S1 subunit contains the receptor-binding domain (RBD), which binds to angiotensin-converting enzyme 2 (ACE2) on the host cells (14). The S2 subunit facilitates fusion between the viral envelope and the host cell membrane through conformational changes in heptad repeat (HR1 and HR2) regions, forming a fusogenic core (15). With only 20–27% sequence identity to other coronaviruses, the S protein is a key therapeutic target. Similar fusion mechanisms are seen in influenza (Hemagglutinin, HA), Ebola (Ebola virus glycoprotein), HIV (HIV glycoprotein 160, Env), and paramyxovirus (Hemagglutinin-neuraminidase, HN).

### 2.2 Nucleocapsid protein

The N protein aids in viral genome packaging, replication, and immune evasion by disrupting interferon (IFN) responses. It is highly conserved and immunogenic (16), making it a viable diagnostic and therapeutic target. Monoclonal antibody CoV396 binds efficiently to its N-terminal domain (NTD) through hydrophobic interactions and hydrogen bonds (17). Inhibitors targeting N-NTD RNA binding and the C-terminal domain’s dimerization may have therapeutic potential.

### 2.3 Envelope protein

The E protein (~75 aa) is a 8.5 kDa cationic viroporin involved in virus assembly, budding, release and endoplasmic reticulum–Golgi intermediate compartment (ERGIC) membrane channel formation (18). Its deletion reduces virulence, and mutations affecting ion channel activity reduce pathogenicity, making protein E a potential candidate for antiviral strategies and vaccine design.

### 2.4 Membrane protein

M is the most abundant structural protein, critical for virion shape and assembly. It interacts with all other structural proteins (19) and impedes host immune responses by antagonistic interactions with IFNs and by inhibiting Nuclear Factor-Kappa B (NF-κB) signaling, lowering the production of COX-2, a key inflammatory protein. Glycosylation and interaction with IkB kinase beta (IKKβ), thereby inhibiting the growth of an IKK signalosome (20), lowers NF-κB activity, influences inflammation and aids in immune evasion. Its immunogenicity and capacity to elicit neutralizing antibodies and cytotoxic T-lymphocyte (CTL) responses (21), support its potential as a vaccine or therapeutic target. Recently, molecular dynamics simulations have identified potential drugs such as remdesivir, targeting the M protein besides RNA-dependent RNA polymerase (RdRp), though further validation is needed (22).

### 2.5 Non-structural proteins

SARS-CoV-2 encodes 15 non-structural proteins (nsp1–nsp16 excluding nsp11) that play key roles in replication, transcription, and immune evasion and may serve as potential therapeutic targets (**Table 1**). These proteins are encoded by open reading frame (ORF)1a/1b regions of the viral genome, and translated as polyprotein that is cleaved by viral proteases. nsp11 (13 aa) is the shortest peptide expressed at the end of ORF1a (**Figure 2**).

**Table 1:** Targeted drugs against SARS-CoV-2 proteins (mediating pathogenesis) and their immuno-epigenetic modulatory effects.

Viral protein targets	Mechanism of pathogenesis	Approved drugs	Immuno-epigenetic relevance of drugs	References
Non-structural protein (nsp)1 (Host shut-off factor)	Host translation regulation by hijacking the translation of host genes (40S ribosome)	Glycyrrhizinic acid, Lobaric acid, Garcinolic acid, Tirilazad	Promotes translation of ISG and MHC-I expression, favors innate and adaptive immunity	(209–212)
nsp2	Facilitates viral replication, host immune regulation, mitochondrial biogenesis, and endosomal transport	Nigellidine	Inhibits viral replication/transcription, FAS-TNF death signal via TNFR 1/2 blocking; no epigenetic effect	(213–215)
nsp3 (PLpro)	Plays a critical role in viral replication and suppression of host immune response by blocking viral polyprotein cleavage and host deubiquitination	Mycophenolic acid	Promotes host antiviral signaling and destabilizes epigenetic repressors	(80, 86, 216)
nsp4	Promotes viral replication	Suvorexant, Eribulin	Dampens stress-induced epigenetic suppression of IFN genes	(182, 217)
nsp5 (3CLpro/ Mpro)	Plays an essential role in viral replication and maturation of nsps	Enanta, Chloroquine, Elbasvir, Cannabisin A, Nirmatrelvir (Paxlovid)	Inhibits viral replication by impairing nsp-mediated chromatin silencing of IFN loci	(218, 219)
nsp6	Induces the formation of a hybrid pre-autophagosomal structure, prevents delivery of virions to host lysosomes	Dextromethorphan, Haloperidol	–	(220)
nsp7	Promotes RNA-dependent RNA polymerase function of nsp12 for viral replication	Vitamin B12	Prevent viral hijacking of host transcriptional regulators and epigenetic suppressors	(29, 221–223)
nsp8	Along with nsp7, promotes RNA-dependent RNA polymerase function of nsp12 for viral replication	Folic acid	Prevent viral hijacking of host transcriptional regulators and epigenetic suppressors, methylates ACE2	(29, 224–226)
nsp9	It is a homodimer involved in formation of the replication and transcription complex (RTC) and plays a significant role in viral replication	Azithromycin, Imidazolium salt	Restores disrupted ncRNA networks	(227, 228)
nsp10	The zinc-finger structure plays a significant role in viral RNA synthesis	Tegobuvir, Sonidegib, Siramesine, Antrafenine, Bemcentinib, Itacitinib, Phthalocyanine	Restores RIG-I/MDA5 sensing, which is epigenetically modulated via histone methylation at PRR loci	(229, 230)
nsp11	Inhibits IFN response, promotes viral replication by regulating endoribonuclease	–	–	(33, 34)
nsp12 (RdRp)	It is the core component of RTC playing a pivotal role in viral proliferation and host immune regulation	Cefuroxime, Surami, Remdesivir, Molnupiravir	Drives epigenetic reprogramming of mucosal immune cells resulting in ISG expression, IFN stimulation	(231–233)
nsp13	It has helicase and RNA 5′-triphosphatase activity thus participates in unwinding duplex DNA or RNA during RNA replication in an ATP-dependent manner	Itraconazole, Saquinavir, Dabigatran, Canrenoic acid	Restores epigenetic checkpoints critical for IFN signaling	(234–236)
nsp14	It removes both mis-incorporated nucleotides and nucleotide analogs from the nascent RNA, making viruses that encode nsp14 prone to nucleotide analog-based antiviral resistance	Aurintricarboxylic acid, Disulfiram, Ebselen, Ribavirin	Reverses epigenetic silencing of host mRNA, destabilizes viral mRNA with host-like cap	(237–240)
nsp15 (EndoU)	Cleaves 5′-polyuridine tracts in negative-strand RNA and prevents the activation of host PRR, MDA5-mediated immune response, acts as IFN antagonist	Congo red, Tipiracil	Epigenetic activation of cytokine and ISG genes	(241–244)
nsp16	It is a heterodimer that plays a fixed role in the S-adenosyl methionine (SAM)-binding pocket of methyl transferase, promoting cap methylation of viral mRNAs	Bictegravir, Dolutegravir, Sinefungin	Exposes viral RNA to PRRs via demethylation, promotes ISG activation	(245–247)
Accessory proteins	They are interspaced between or within the viral structural protein genes, and have some genus or species specificity	–	–	(43)
Open reading frame protein (ORF)3a	It is the largest accessory protein in SARS-CoV-2 which promotes viral pathogenicity by disrupting the cellular physiology of the host cell	Viroporin inhibitors -Amantadine, Rimantadine	–	(248, 249)
ORF6	Dampens IFN signaling, blocks nuclear import of STAT1 and mRNA export	Selinexor, IFN inducers	Induces IFN transcription and nuclear translocation of antiviral genes, makes histone accessible at ISG promoters	(45, 250, 251)
ORF7a	It is a type-I transmembrane protein that triggers blocking of IFN signaling and apoptosis of infected host cells	Proteins	–	(252, 253)
ORF8	It is a multifunctional protein that induces host cell apoptosis, suppresses the host innate immune response, plays a role as an antagonist of type-I IFN, downregulates MHC-I	Proteins	Promotes NLRC5-mediated MHC-I gene activation and adaptive immune recognition	(49, 254, 255)
ORF9b	It has a tent-like shape formed by two intertwined monomers. It promotes the transfer of preproteins to mitochondria and suppresses host’s IFN-I responses	Antibodies	–	(51, 256)
ORF10	Hijacks ubiquitinylation machinery	–	–	(257)
S	Mediates viral entry via ACE2 receptor binding and membrane fusion	Bebtelovimab, Camostat	Regulates ACE2 expression via DNA demethylation, stabilizes ISG expression, reduces TLR-mediated histone acetylation of pro-inflammatory genes	(258)
E	Helps in viral assembly, ion transport, and inflammasome activation	Amantadine, hexamethylene amiloride, Viroporin inhibitors, ion channel blockers	Normalizes stress-responsive epigenetic modifiers and modulate NF-κB activation-linking to chromatin remodeling, suppress epigenetically primed inflammatory circuits via reduced inflammasome activity, rebalance lncRNA networks that control transcription of antiviral and immune genes	(259)

nsp1 (Host Shutoff Factor) suppresses host gene expression via 40S ribosome binding, leading to mRNA degradation. This host shutoff mechanism inhibits antiviral responses (23). Drugs disrupting nsp1-ribosome interaction may restore host protein synthesis and innate immunity. nsp2 interacts with host proteins like prohibitin, impacting key mitochondrial functions, lipid homeostasis and innate immunity (24). Further studies are needed to assess its potential as a drug target. nsp3 (Papain-like protease, PLpro) has protease activity, cleaving the viral polyprotein to release individual nsps. It also deubiquitinates and deISGylates host proteins, subverting immune responses (25). PLpro inhibitors, such as GRL-0617, are being investigated for their antiviral effects. nsp4/6 remodel host membranes to form double-membrane vesicles (DMVs), which serve as replication organelles for the virus (26). Drugs that disrupt DMV formation could inhibit viral replication. nsp5 (Main protease, 3CL{chymotrypsin-like}pro/Mpro) cleaves viral polyproteins at 11 distinct sites, making it crucial for viral maturation (27). 3CLpro inhibitors, such as nirmatrelvir, have shown efficacy in reducing viral replication. nsp7/8 forms RNA primase complex and aids RdRp, facilitating viral genome replication (28). They also curb IL-8 activation and subsequent recruitment of neutrophils, facilitating viral replication (29). Drugs that interfere with nsp7/8 complex could inhibit replication. nsp9 binds single-stranded (ss) RNA, aiding viral RNA synthesis (30). Inhibitors that disrupt RNA binding may interfere with replication. nsp10 acts as a cofactor for nsp14/16, enhancing their exonuclease and methyltransferase activities (31, 32). Drugs targeting nsp10 interface could suppress multiple enzymatic functions. nsp11 suppresses host’s immunity by inhibiting IFN response (33). It also regulates endoribonuclease, essential for viral replication (34). nsp12 (RdRp) catalyzes RNA synthesis, using nsp7/8 as cofactors (35). RdRp is targeted by remdesivir/molnupiravir, disrupting viral RNA synthesis (36). nsp13 (helicase) unwinds RNA secondary structures during replication and transcription (37). Helicase inhibitors, such as bananins, have shown promise in preclinical studies (38). nsp14 has proofreading exonuclease activity, ensuring replication fidelity, and guanine-N7 methyltransferase activity for RNA capping (39). Inhibitors targeting nsp14’s exonuclease or methyltransferase domains may reduce viral viability (40). nsp15 (endoribonuclease, NendoU) cleaves viral and host RNA, and evades immune detection by degrading viral RNA intermediates (41). NendoU inhibitors could disrupt immune evasion and inhibit viral RNA synthesis. nsp16 (2′-o-methyltransferase) modifies viral RNA to mimic host mRNA, evading immune detection. Inhibitors of nsp16 methyltransferase activity could restore immune recognition (42).

### 2.6 Open reading frames as accessory proteins

SARS-CoV-2 encodes several accessory proteins (e.g., ORF3a, ORF6, ORF7a, ORF8, ORF10), which are not required for replication but modulate host responses and pathogenesis (**Table 1**) (43). ORF3a forms ion channels, induces apoptosis and inflammation and antagonizes IFNs. Ion channel blockers could inhibit its pathogenic effects (44). ORF6 interferes with nuclear import by binding to karyopherin/importin, suppressing IFN signaling (45). Inhibitors that restore nuclear transport could counteract ORF6 activity. ORF7a antagonizes IFN responses, promotes apoptosis of infected host cells (46, 47). It inhibits autophagosome-lysosome fusion, thereby promoting viral pathogenesis (48). Blocking ORF7a interactions with host proteins may reduce immune suppression. ORF8 downregulates major histocompatibility complex I (MHC-I), aiding the virus to evade cytotoxic T cells (49). ORF8 inhibitors could enhance immune clearance of infected cells (50). ORF9b suppresses interferon response, inhibiting antiviral signaling (51). ORF10 is poorly characterized and may influence ubiquitination pathways mediating proteosomal degradation (52). Further studies are required to identify specific therapeutic targets.

## 3 Specific interplay of the SARS-CoV-2 at the mucosal interface and replication in the host

**Figure 3:**
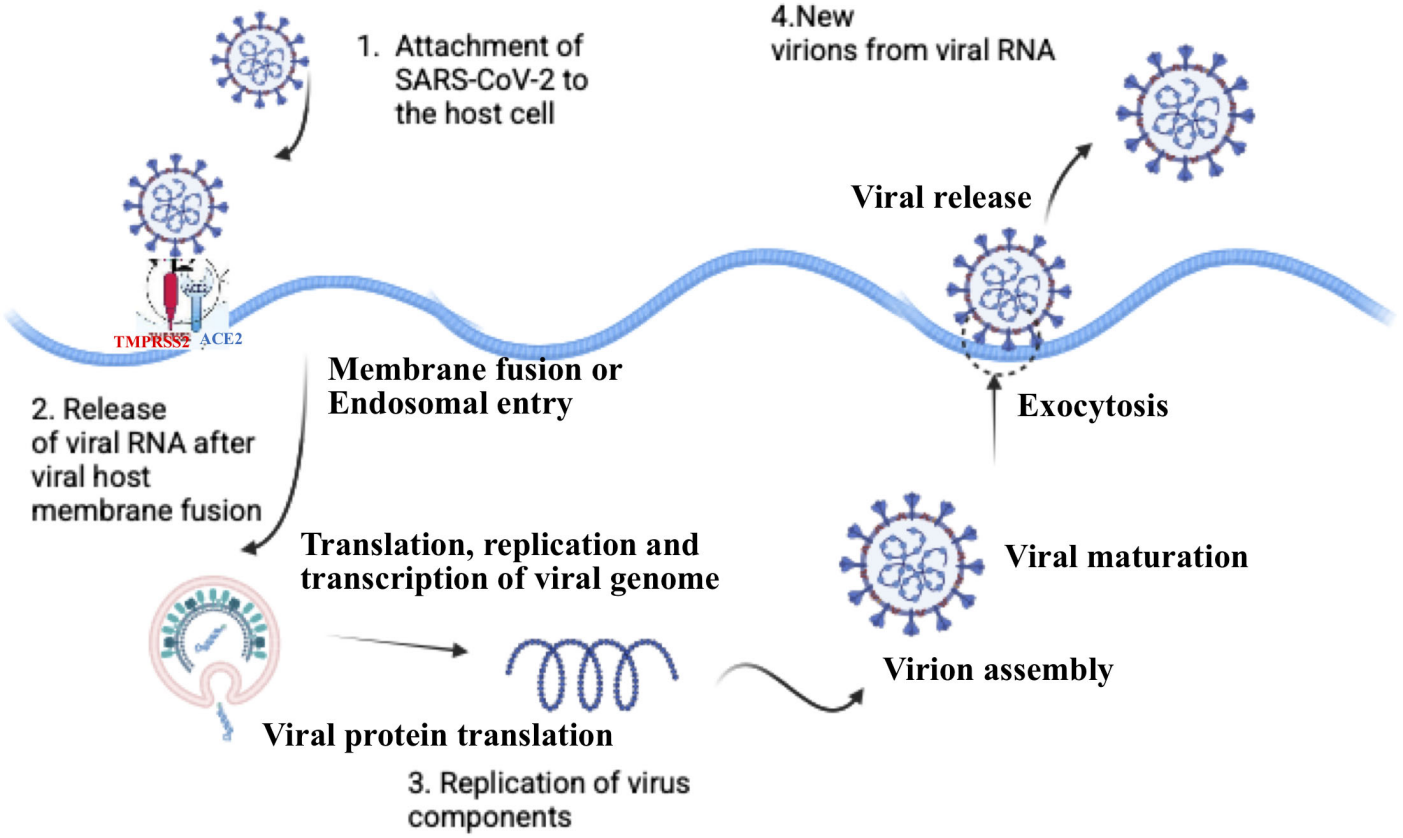
Life cycle of SARS-CoV-2. The transmembrane protein angiotensin-converting enzyme 2 (ACE2) on human host cells is the principal receptor for the spike (S) protein of SARS-CoV-2 virus and facilitates entry of the virus into the human cells. Numerous human cell types, including the epithelial lining of the lungs, colon, liver, kidney, heart, and other tissues, have the ACE2 receptor proteins on their surface. Following receptor binding, the virus enters the cells by acid-dependent proteolytic cleavage of the S protein by TMPRSS2 or other proteases. TMPRSS2 is principally expressed in the airway epithelial cells and vascular endothelial cells. This leads to viral and host cell membrane fusion and release of the viral genomic RNA into the host cytoplasm. The viral RNA initiates translation of co-terminal polyproteins that are subsequently cleaved into nonstructural proteins (Nsps) by Mpro and PLpro. Several Nsps interact with Nsp12 (RdRp) to form the replicase-transcriptase complex (RTC), which is responsible for the synthesis of full-length viral genome (replication) and sub-genomic RNAs (transcription). The viral structural proteins are expressed and translocated into the endoplasmic reticulum (ER). The nucleocapsid (N) protein-encapsulated genomic RNA translocates with the structural proteins into the ER-Golgi intermediate compartment (ERGIC) for virion assembly. The newly synthesized virions are budded through the cell membrane and exocytosed, releasing the mature virions.

SARS-CoV-2 primarily invades the human host via the respiratory tract. The mucosal surfaces of the nasal and upper respiratory epithelium serve as the primary portals of viral entry and early replication. The mucosal interface is a highly specialized immunological niche that plays a crucial role in determining the clinical outcome of infection. The replication cycle of SARS-CoV-2 is a tightly regulated process involving viral entry, RNA replication, and virion assembly, all while evading host immune defenses (**Figure 3**). The virus-host dynamics at the mucosal level is discussed here with an emphasis on entry mechanisms and mucosal immune responses.

### 3.1 Viral entry and tropism at the mucosal interface

SARS-CoV-2 initiates infection at the nasal mucosa, where respiratory epithelial cells, particularly goblet and ciliated cells, express high levels of ACE2 and TMPRSS2 (53). The fusogenic S glycoprotein binds ACE 2 via RBD of the S1 subunit, facilitating viral attachment (54). Proteolytic activation/priming by host proteases, TMPRSS2 and furin at the S1/S2 and S2’ sites induces conformational change and enables membrane fusion and entry of viral RNA into the host cytoplasm (55). In the absence of TMPRSS2, alternative endocytic routes, such as clathrin-mediated endocytosis, allow internalization, although with reduced efficiency (56). Thus, individuals with diminished TMPRSS2 expression may still be susceptible to infection through endosome-dependent pathways (57). Experimental models reveal that loss or reduction of ACE2 worsens lung injury after viral infection, likely due to unchecked angiotensin II activity via angiotensin II type 1 receptor (AT1R), leading to inflammation, vasoconstriction and fibrosis (58, 59). Clinically, this RAAS imbalance correlates with severe COVID-19 outcomes, indicating that while ACE2 downregulation may limit viral entry, it risks exacerbating disease by disrupting homeostatic pathways (Wang X et al., 2025). Early viral replication in the nasopharynx contributes to high transmissibility. Mutation like D614G in the S protein enhances ACE2 binding affinity, facilitating more efficient mucosal colonization (60). Understanding these mechanisms has informed the development of entry inhibitors and monoclonal antibodies (61).

### 3.2 Synthesis of viral RNA and structural proteins

Once inside the cytoplasm, the viral positive-sense RNA (+ssRNA) serves directly as mRNA, initiating translation by hijacking the host’s ribosomes to produce viral proteins. ORF1a and ORF1b, comprising about 75% of the viral genome, are translated into polyproteins pp1a and pp1ab, which are cleaved by viral proteases, PLpro and 3CLpro (main protease, Mpro), into nsps that form the replication-transcription complex (RTC). The key components include nsp12 (RdRp) that synthesizes new viral RNA, nsp13 (helicase) that unwinds RNA, nsp14 (exonuclease) that ensures replication fidelity (28). Replication occurs within double-membrane vesicles (DMVs) derived from the ER (62, 63). Initially, RTC synthesizes negative-sense RNA (-ssRNA) as a template for generating multiple new genomic (+ssRNA) copies (64). Simultaneously, discontinuous transcription produces sub-genomic RNAs (sgRNAs), encoding structural proteins (S, M, E, N) and accessory proteins (65). The RNAs are translated to form components needed for new virions.

### 3.3 Virion assembly

Structural proteins synthesized in the ER are transported to ERGIC wherein, N protein packages the new viral RNA into nucleocapsids (66). While S, M, and E proteins are embedded into the ERGIC membrane of budding virions that assemble within this compartment. Instead of using the classical secretory pathway, SARS-CoV-2 exits via lysosomal trafficking, helping it evade host immune detection (62). Fully assembled virions are released by exocytosis, allowing infection of neighboring cells.

### 3.4 Mucosal immune responses to SARS-CoV-2

The mucosal surfaces of the respiratory tract serve as the primary entry point and battleground for SARS-CoV-2. As the first line of host defense, the mucosal epithelium and associated immune components orchestrate a dynamic interplay with the virus, influencing disease outcomes and transmission. Understanding how SARS-CoV-2 exploits, modulates, and is countered at this critical barrier is essential to unraveling its pathogenesis and informing mucosal vaccine strategies. Herein, the specific molecular and immunological interactions between SARS-CoV-2 and the mucosal interface is explored, highlighting mechanisms of viral entry, immune evasion, and local immune responses.

#### 3.4.1 Mucosal entry and tropism

##### 3.4.1.1 Receptor expression at the mucosa

The nasal epithelium, particularly goblet and ciliated cells, exhibits high levels of ACE2 and TMPRSS2 expression, facilitating viral entry (67). The virus binds via the S protein’s RBD, initiating endocytosis or membrane fusion.

##### 3.4.1.2 Preferential infection of upper airways

Early infection and replication are most robust in the nasopharyngeal mucosa, a site enriched in secretory IgA and innate immune sensors that stand as the sentinels of these portals (68). This tropism contributes to the high transmissibility of SARS-CoV-2 and underscores the significance of the mucosal immune landscape in controlling initial viral spread.

#### 3.4.2 Innate immunity

Upon infection, alveolar epithelial cells activate pattern recognition receptors (PRRs), including retinoic acid-inducible gene-I (RIG-I), MDA5 and TLR3, and respond rapidly by releasing type I and III IFNs, IL-6 and chemokines such as CXCL10. These responses orchestrate the recruitment of neutrophils, monocytes, and NK cells (69). However, SARS-CoV-2 dampens IFN signaling via nsps such as nsp1, nsp6, allowing early immune evasion (70).

#### 3.4.3 Secretory IgA and adaptive responses

Mucosal B cells produce SARS-CoV-2-specific secretory dimeric IgA (sIgA), which is transported across the epithelium via the polymeric immunoglobulin receptor (pIgR) (71). sIgA neutralizes virus particles in the airway lumen, blocking further cell entry at the mucosal interface. SARS-CoV-2-specific sIgA is detectable in saliva and nasal washes post-infection or mucosal vaccination and correlates with local viral control.

#### 3.4.4 Mucosal-associated invariant T cells, tissue-resident memory-Lsike T cells and alveolar macrophage

MAIT and TRMs are innate-like T cells that are activated early in infection and are abundant in the airway mucosa. TRMs and AMs play central roles in mucosal defense. TRMs provide localized, rapid adaptive immune responses upon re-infection. SARS-CoV-2 may dysregulate MAIT cell function, leading to hyperinflammation of the mucosa (72).

The mucosal interface represents a critical site for early SARS-CoV-2 interaction, immune modulation, and pathogenesis. The interplay between the virus and mucosal immune elements determines both infectivity and disease progression. A deeper understanding of these mechanisms will inform the rational design of mucosal-targeted therapeutics and next-generation vaccines.

## 4 Epigenetics and COVID-19

Investigations in epigenetics have demonstrated that DNA and RNA viruses can adopt antagonistic regulatory roles by modifying host metabolism and gene expression, thereby creating an environment conducive to viral propagation and replication (73). The viruses interact with host cellular machinery, including chromatin remodeling and gene regulation systems, by modulating DNA methylation, histone modifications, and miRNA expression. The epigenetic processes are central to understanding COVID-19 pathogenesis, as SARS-CoV-2 induces various epigenetic changes to manipulate host’s epigenome, enhance viral replication, and suppress host’s immune defenses (74). Coronaviruses specifically promote epigenetic changes by interfering with the host’s antigen presentation and inhibiting the activation of interferon (IFN)-responsive genes (75). The epigenetic modifications such as DNA methylation of ACE2 or IFN genes, histone modifications in airway epithelial cells or resident immune cells specifically impact viral entry, replication, and immune responses (innate and adaptive) at mucosal sites.

**Figure 4:**
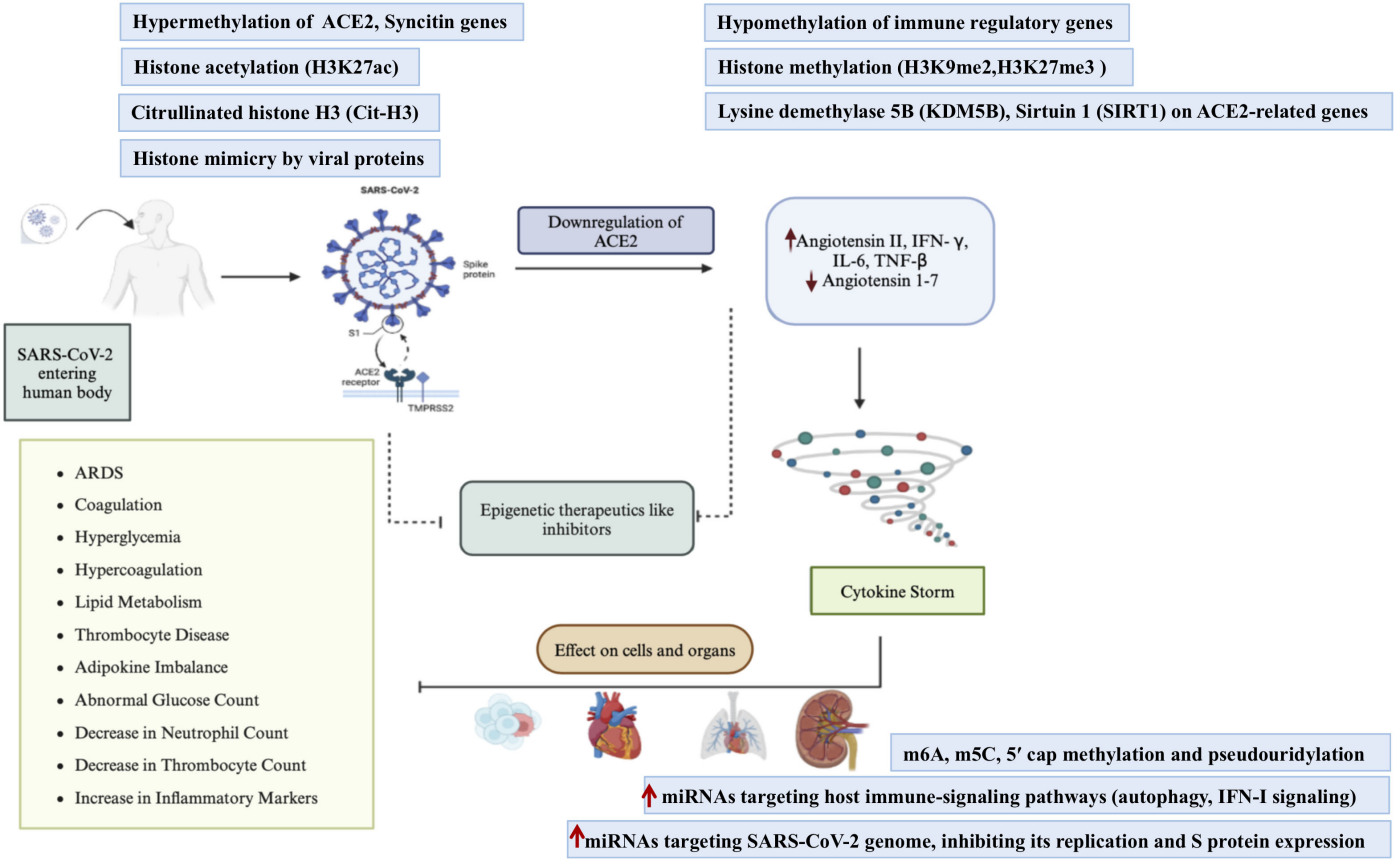
Epigenetic treatments to inhibit the molecular mechanisms driving the cytokine storm in SARS-CoV-2 infection. Such treatments may target key proteins like Furin, ACE2, and TMPRSS2. The diagram illustrates how epigenetic modulation may reduce the overproduction of pro-inflammatory cytokines such as tumor necrosis factor (TNF), interleukin (IL), and interferon (IFN), thereby downregulating the cytokine storm associated with severe disease outcomes. ↑ denotes upregulation, and ↓ denotes downregulation.

SARS-CoV-2 like many RNA viruses, does not possess its own DNA-based epigenetic machinery or traditional histone-based chromatin, as its genome consists of a single-stranded RNA. However, emerging evidence suggests that SARS-CoV-2 exhibits virus-associated epigenetic-like modifications that influence its replication, virulence, immune evasion and interaction with the host. These changes are primarily mediated through RNA modifications, structural changes, and interactions with the host epigenetic machinery. Understanding these epigenetic-like processes provides critical insights into viral pathogenesis and may pave the way for innovative antiviral therapies. The epigenetic-like changes induced in SARS-CoV-2 during infection are illustrated in **Figure 4** and discussed below.

### 4.1 DNA methylation alterations in coronavirus

DNA methylation plays a pivotal role in regulating gene expression. It involves the addition of methyl groups to cytosine residues in CpG islands by DNA methyltransferases (DNMTs), leading to gene silencing. Specifically, methylation in the promoter region obstructs the binding of transcription factors resulting in transcriptional repression. SARS-CoV-2 infection alters host DNA methylation patterns, contributing to disease progression and severity (76). This suggests a potential risk factor for COVID-19 associated with the host epigenome. Recent studies have shown altered DNA methylation patterns in COVID-19 patients, with significant changes observed at 172 kidney-specific sites and 49 heart-specific sites within seven days of infection (77). Additionally, genome-wide DNA methylation studies in severe COVID-19 patients suggest accelerated biological aging compared to those with milder symptoms. Even patients with mild COVID-19 symptoms exhibited notable signs of increased aging compared to healthy individuals (78). Understanding methylation changes could pave the way for novel therapies, including DNA methylation inhibitors, to restore normal gene expression.

#### 4.1.1 Global methylation changes

SARS-CoV-2 infection causes global hypomethylation, impacting genes involved in immune regulation and viral entry (79). The dysregulation of DNA methylation can result in overactivation of pro-inflammatory cytokine genes, contributing to severe COVID-19 complications such as cytokine storm (80). This can lead to acute respiratory distress syndrome (ARDS) and other complications. Persistent hypomethylation post-infection may lead to chronic immune dysregulation and increased susceptibility to long COVID (81).

#### 4.1.2 ACE2 methylation and age-related epigenetic changes

Age-related DNA methylation alterations in airway epithelial cells have been reported to occur near the transcription start site of ACE2. Utilizing the Illumina DNA methylation array, samples representing various biological ages have been found to exhibit hypomethylation at a CpG site close to the ACE2 transcription start site (TSS200 region) as a function of aging (82). Previous research has demonstrated that aging is associated with global downregulation of DNA methylation, leading to distinct methylation patterns in aging, inflammatory, and immune response genes. This aligns with the concept of epigenetic aging, where certain genes become more active while others become less so. Consequently, increased expression of ACE2 in lung tissue among older adults may enhance their susceptibility to viral infections, including COVID-19. In contrast, the ACE2 gene is typically hypermethylated in the organs and tissues of children, rendering it effectively silenced. This phenomenon may explain the heightened sensitivity of older individuals to symptomatic SARS-CoV-2 infection compared to younger populations (83). Hypomethylation near the ACE2 gene transcription start site increases ACE2 expression in older adults, enhancing their susceptibility to SARS-CoV-2. In contrast, children exhibit hypermethylation of ACE2 and other viral entry-related genes, which may contribute to milder disease presentations.

#### 4.1.3 Gene-specific methylation

SARS-CoV-2 exploits genes like syncytin-1 and syncytin-2, typically hypermethylated in tissues other than placenta, to promote viral entry. During infection, these genes become hypomethylated, facilitating syncytium formation and viral replication (84). Genes associated with the innate immune responses, such as those involved in interferon signaling, can also be affected. Hypomethylation may enhance their expression but can lead to dysregulated immune responses. Conversely, some antiviral genes may be downregulated due to increased methylation, impairing the host’s ability to mount an effective immune response against the virus.

### 4.2 Histone modifications and COVID-19

Histone modification serves as another critical mechanism of epigenetic regulation influencing the severity of COVID-19. Histone modifications regulate gene expression by altering chromatin structure. SARS-CoV-2 impacts histone acetylation, methylation, phosphorylation, ubiquitination and other modifications which influence the accessibility of DNA to transcription factors and other regulatory proteins, to modulate host immune responses (80). Dysregulated histone modifications enhance susceptibility to viral replication and severe inflammation.

#### 4.2.1 Histone acetylation and deacetylation

Histone acetyltransferases (HATs) add acetyl groups to lysine residues on histones, neutralizing the overall positive charge of histones, thereby relaxing chromatin and promoting transcription by facilitating access for transcription factors to target genes. Histone deacetylases (HDACs) remove acetyl groups, condensing chromatin and repressing transcription. During Coronavirus infection, histone acetylation and deacetylation function as molecular switches that toggle gene expression, primarily regulated by HATs and HDACs (85). In general, histone acetylation relaxes the chromatin, allowing transcription factors to reach DNA and start the transcription of genes (86). Increased acetylation at ISG promoters during SARS-CoV-2 infection speeds up their activation, which is necessary to mount a potent antiviral state (87). The ISGs encode proteins that enhance immune signaling, promote antigen presentation, and obstruct viral replication. In contrast, by reducing acetylation at these loci through manipulation of the host’s epigenetic machinery, the virus can weaken the IFN response and increase its rate of replication (87). Targeting epigenetic regulators like HDACs and HATs could restore balanced gene expression and improve immune responses. Pathway enrichment analyses have shown that ACE2-related genes are also regulated by histone acetylation marks, such as acetylation of lysine 27 (H3K27ac) on histone H3 (88).

#### 4.2.2 Histone H3 methylation

Besides histone acetylation, alterations in H3 lysine methylation (such as H3K4me3) impact chromatin accessibility and gene expression, including those involved in immune signaling. Histone methylation can either activate or repress gene transcription, depending on the particular residue and methylation status (mono-, di-, or tri-methylation). For instance, active transcription is linked to the methylation of histone H3 on lysine 4 (H3K4me3), and its presence at loci encoding pro-inflammatory molecules like TNF-α and IL-6 and cytokine gene loci like those encoding IFNs can increase their expression during infection (89). However, methylation at H3K27 (H3K27me3) is associated with gene silencing and has been observed to inhibit antiviral genes in certain situations, which may help SARS-CoV-2 evade the immune system (89).

Thus, transcription-repressing histone modifications, such as H3K9me2 and H3K27me3 in promoter of immune responsive genes have been reported to inhibit antiviral immune response (90). ACE2-related genes are regulated by lysine demethylase 5B (KDM5B) alongside specific histone methylation and acetylation marks, including mono-methylation (me) and trimethylation (me3) of lysine 4 (K4) on histone H3 (88). KDM5B modulates chromatin accessibility by repressing active chromatin marks, including di- and trimethylation of H3K4, which are important for DNA repair and transcriptional activation. Additionally, it has been indicated that inhibition of KDM5B can trigger an IFN response, enhancing resistance to DNA and RNA viral infections. This suggests a potential role for KDM5B as a target for COVID-19 prevention.

Frontline sensors that identify viral RNA include PRRs, such as RIG-I and TLR3, which set off-signaling cascades that ultimately result in the production of IFN (91). Histone modifications also control the accessibility of the genes that encode these receptors (91). In order for these receptors to be expressed at high enough levels to quickly identify viral components, proper acetylation and activating methylation marks are necessary. Nevertheless, it has been documented that SARS-CoV-2 disrupts these epigenetic markers, resulting in decreased PRR expression, postponed virus detection, and a weakened early immune response (6).

#### 4.2.3 Regulators of ACE2 expression

Severe COVID-19 cases have been associated with elevated lung ACE2 expression, particularly in patients with comorbidities. Analysis of 700 human lung transcriptomes exhibiting high ACE2 expression in conjunction with underlying conditions, has identified HAT1, HDAC2, KDM5B and Sirtuin 1 (SIRT1), a NAD-dependent HDAC, as potential regulators of ACE2 expression (92). SARS-CoV-2 interferes with HDAC2 nuclear localization via non-structural protein nsp5, disrupting immune responses.

#### 4.2.4 Histone Citrullination

NETosis, a controlled type of cell death, is essentially a form of innate immunity, that relies on entangled trap-like aggregates of DNA, histone and proteins termed ‘neutrophil extracellular traps’ (NETs). A specific marker of NETs, citrullinated histone H3 (Cit-H3) has been found to be elevated levels in blood of SARS-CoV-2 patients (93).

#### 4.2.5 Histone mimicry by viral proteins

The E protein and other SARS-CoV-2 nsps such as nsp5 and nsp8 contain motifs that mimic histone tails, allowing them to bind to bromodomain proteins (BRD2 and BRD4). This competitive binding disrupts the recognition of acetylated histones, impairing the transcription of interferon-stimulated genes (ISGs) and other immune defense mechanisms (94).

### 4.3 miRNAs and SARS-CoV-2

miRNAs are small, non-coding RNAs, usually about 22 nucleotides long, that regulate gene expression after transcription by targeting complementary sequences on specific mRNAs, leading to their degradation or translational inhibition. SARS-CoV-2 disrupts host miRNA profiles, influencing immune responses and viral replication (95). Profiling miRNA expression may serve as a biomarker for disease severity and guide personalized treatments. Modulating miRNA expression offers a promising avenue for therapeutic interventions to enhance antiviral responses.

#### 4.3.1 Host miRNA profiling

A regulatory triangular network is established among the host, the virus, viral-encoded miRNAs, host miRNAs, and their respective mRNA targets, which collectively influence the severity of infection. Certain miRNAs are upregulated in response to infection, either suppressing or facilitating viral replication (miRNA-155-5p, miRNA146a-3p, miRNA-29a-3p, miRNA-98-3p, miRNA-32-5p, miRNA-1246, miRNA-2392, miRNA-423-3p, etc.) while miRNA 144 and miRNA 1823 are downregulated (96). In older adults, the diminished levels of host defense miRNAs, in particular exosomal miRNAs may contribute to the heightened severity of COVID-19 and the risk of mortality (97). In a profiling study involving 67 SARS-CoV-2 patients across 24 countries, Khan et al. found that certain induced miRNAs can either inhibit viral expression or promote viral replication (98). An array of long non-coding RNAs (lncRNAs) and miRNAs hijacked by SARS-CoV-2 facilitating immune evasion by SARS-CoV-2 has been recently reviewed (99). Their research suggests that miRNAs could serve as potential therapeutic targets for addressing complications arising from COVID-19.

Host miRNAs such as hsa-let-7a, hsa-miRNA-129, and hsa-miRNA-125a-5p directly target viral RNA, while others influence immune and inflammatory pathways. Moreover, a separate study utilizing gene ontology and nucleotide similarity analysis of five SARS-CoV-2 genomes identified 22 potential viral miRNAs that correspond to 12 human miRNAs, indicating that interactions between human host miRNAs and the viral genome could impact host pathways in ways that remain to be fully elucidated regarding the virus’s pathogenic mechanisms (100).

#### 4.3.2 miRNA and epitranscriptomic modifications in the SARS-CoV-2 genome

SARS-CoV-2 encodes its own miRNAs, which inhibit host antiviral proteins thereby facilitating SARS-CoV-2 infection by modulating immune signaling pathways, including Janus kinase (JAK) 1 and 2, as well as various signal transducers and activators of transcription (STAT) genes (3, 4, 5B, and 6), along with suppressor of cytokine signaling (SOCS) genes. For instance, miRNAs such as hsa-let-7a, hsa-miRNA-129, hsa-miRNA-125a-5p, hsa-miRNA-101, hsa-miRNA-378, hsa-miRNA-23b, and hsa-miRNA-380-5p may directly target the SARS-CoV-2 genome and inhibit its replication and S protein expression (101).

Epitranscriptomics focuses on the chemical changes that occur in RNA molecules after transcription, without altering their basic nucleotide sequence. These modifications affect RNA’s fate by regulating splicing, nuclear export, translation, and degradation (102). Over 150 RNA modifications have been identified, but N6-methyladenosine (m^6^A) is the most common and well-studied internal change in eukaryotic mRNAs and non-coding RNAs. It plays a vital role in regulating gene expression and is now recognized as important for controlling antiviral immunity. Writers such as METTL3, METTL14, and WTAP add m6A marks; erasers like FTO and ALKBH5 remove these marks (103) while readers, such as YTHDF1–3 and YTHDC1, interpret them (104). This enzyme system allows for rapid and reversible changes in RNA metabolism when environmental conditions shift, including viral infections.

SARS-CoV-2 leverages multiple forms of post-transcriptional RNA modifications (epitranscriptome) to regulate its viral RNA stability, translation efficiency, and evasion of host immune responses (105). These epitranscriptomic modifications mimic epigenetic regulation typically seen in DNA-based genomes (105). m^6^A modification is the most common alteration found on the viral RNA of SARS-CoV-2 (106). This chemical addition occurs on adenosine residues and is catalyzed by host methyltransferase complexes, including METTL3, METTL14, and WTAP. This alteration enhances viral RNA stability by protecting it from host exonucleases, increases translation efficiency of viral proteins, aiding in effective replication and regulates the interaction of viral RNA with host immune system, enabling immune evasion. m^6^A marks has been reported to reduce the recognition of viral RNA by host pattern recognition receptors such as RIG-I.

Epitranscriptomic 5-methylcytosine (m^5^C) of SARS-CoV-2 RNA has been reported to regulate viral replication and virulence (107). Epitranscriptomic addition of m^5^C to SARS-CoV-2 RNA has been proposed as host’s antiviral strategy (108). Other epitranscriptomic RNA modifications in SARS-CoV2 include 5′ cap methylation and pseudouridylation (Ψ). The 5′ cap of the SARS-CoV-2 RNA undergoes methylation modifications, mimicking host mRNA, to avoid degradation by host exonucleases and evade immune detection (105). This modification ensures efficient translation initiation of viral proteins. The introduction of pseudouridine into viral RNA by host enzymes enhances RNA folding, stability and translation efficiency, and viral replication (106).

### 4.4 Structural and epigenetic-Like RNA modifications

#### 4.4.1 RNA secondary and tertiary structures

The SARS-CoV-2 genome forms intricate secondary structures, such as stem-loops, pseudoknots, and G-quadruplexes. These structures act as regulatory elements, influencing viral replication, translation, and packaging (109). G-quadruplex structures, in particular, are hotspots for host protein binding, which facilitates viral replication.

#### 4.4.2 Host-driven RNA editing

Host enzymes such as adenosine deaminase acting on RNA (ADAR) and apolipoprotein B mRNA-editing enzyme complex (APOBEC) induce A-to-I (adenosine-to-inosine) or C-to-U (cytidine-to-uridine) edits on viral RNA (110). The ability of the viral RNA to undergo host-driven modifications (e.g., A-to-I or C-to-U editing) increases its mutational flexibility, which can lead to the emergence of immune-evasive strains. While some of these edits can be detrimental to the virus by introducing mutations, others are selectively tolerated or even advantageous, enhancing viral adaptation and immune evasion.

## 5 Epigenetic regulation at mucosal barriers: gatekeeping the host response

Epigenetic mechanisms are critical regulators of host-pathogen interactions at mucosal surfaces such as the respiratory, gastrointestinal, and urogenital tracts, where SARS-CoV-2 initiates infection (10). The respiratory mucosa, a primary site of SARS-CoV-2 infection, is equipped with specialized immune and epithelial networks that are subject to dynamic epigenetic regulation. These epigenetic regulatory systems including DNA methylation, histone modifications, and non-coding RNAs, modulate chromatin architecture and transcription without altering the underlying DNA sequence, shaping the host immune landscape (111). Mucosal epithelia represent the first line of defense, where rapid immune activation is delicately balanced with tolerance to commensals and innocuous stimuli (112). This immune equilibrium is tightly governed by epigenetic marks that orchestrate transcriptional programs in epithelial and immune cells exposed to viral invasion (70). Like other respiratory viruses (influenza or RSV), SARS-CoV-2 infection also elicits epigenetically reprogrammed responses that fine-tune the expression of viral entry receptors, ISGs and innate and adaptive immune effectors such as IFN production, chemokine signaling, and immune cell recruitment, influencing disease severity and immune evasion (113).

### 5.1 Regulation of viral entry: DNA methylation of ACE2 and co-factors and mucosal gene expression

SARS-CoV-2 entry into mucosal epithelial cells relies on ACE2, whose expression is governed by both genetic and epigenetic factors (114). DNA methylation at the ACE2 promoter and enhancer regions inversely correlates with ACE2 expression in airway epithelial cells. It suppresses transcription, reducing receptor availability and viral entry but potentially impairing tissue homeostasis, especially within the pulmonary and renal systems (115, 116).

Age, sex, and comorbidities modulate ACE2 expression via methylation-dependent mechanisms, contributing to differential susceptibility across populations (117, 118). Gene expression profiling in humans and mouse models has shown consistently increased abundance of ACE2 mRNA in older males that correlated with higher viral loads and severe outcomes (119). These results highlight the intricate cross talk between genetic and epigenetic processes that regulate receptor abundance and thereby individual susceptibility to SARS‐CoV‐2 infection. ACE2 is also a critical regulator of the renin-angiotensin-aldosterone system (RAAS) which is important for the homeostasis of cardiovascular system and pulmonary function (115). Thus, excessive suppression risks promoting angiotensin II-mediated inflammation and fibrosis (58, 120). Similarly, TMPRSS2, a protease that primes the S protein for membrane fusion, is influenced by DNA methylation status. Alternative proteases like cathepsins and furin provide redundant entry routes, expanding viral tropism via miRNA-106a (121, 122). This epigenetically controlled redundancy underscores SARS-CoV-2’s adaptability in mucosal environments. Studies have shown that hypomethylation of ACE2 in nasal epithelium (especially in smokers and the elderly) enhances its transcription, potentially increasing susceptibility to infection (Corley & Ndhlovu, 2021). Conversely, hypermethylation may restrict entry in individuals with lower risk profiles. Similarly, epigenetic regulation of TMPRSS2 and FURIN via androgen-responsive enhancers or methylation-sensitive regions influences viral activation potential at mucosal surfaces.

### 5.2 Histone modifications in airway epithelia and resident immune cells

SARS-CoV-2 rewires host defenses to facilitate their replication and survival by modifying chromatin structure, and gene accessibility (86). Viral tactics primarily target histone acetylation, methylation, and deacetylation, each of which has a unique function in immune suppression. Histone acetylation and methylation dynamically regulate chromatin accessibility, influencing antiviral gene expression. During infection, SARS-CoV-2 exploits host chromatin-modifying enzymes to suppress ISGs and PRRs (86, 123).

Peptidylarginine deiminases (PADs) catalyze the conversion of specific arginine residues on histone tails into citrulline (90). Hyperactivation of neutrophils in severe COVID-19 leads to formation of neutrophil extracellular traps (NETs), characterized by cell-free citrullinated histone H3 (Cit-H3), a product of PAD4 activity (124). This post-translational modification is typically initiated in response to intracellular calcium signaling and subsequent activation of neutrophils, positioning PADs as central mediators of NET-driven inflammation during SARS-CoV-2 infection (125). Agents like Cl-amidine that inhibit PAD4 can significantly reduce NET release in neutrophils from individuals infected with SARS-CoV-2 (126). PAD4-mediated histone citrullination weakens histone-DNA interactions, promotes chromatin decondensation, NETosis, and triggers inflammatory tissue damage and disease progression in critically ill patients (126, 127). The presence of Cit-H3 is strongly associated with increased counts of circulating platelets, leukocytes, granulocytes, and heightened levels of pro-inflammatory cytokines such as IL-8 (128). It remains unclear whether PAD4 is activated by the direct invasion of neutrophils by the virus or indirectly through the strong pro-inflammatory signals associated with cytokine storms that are common in severe COVID-19 cases (129).

The histone modifications modulate not only neutrophil responses but also suppress antiviral epithelial IFN responses and macrophage activation profiles. TRMs from COVID-19 lungs have been reported to exhibit altered chromatin landscapes, including reduced H3K4me3 at loci encoding IFN-γ and TNF, which correlate with T cell exhaustion and reduced effector functions (130). SARS-CoV-2 infection is associated with epigenetic silencing of IFN genes, especially IFNL1 and IFNB1, in mucosal epithelial and dendritic cells. Increased levels of repressive histone marks such as H3K27me3 and reduced activating marks (H3K4me3, H3K27ac that promote transcription of ISGs) at IFN loci have been detected in infected respiratory tissues, which correlates with blunted type I and III IFN responses (89, 91). This suppression delays or hinders antiviral signaling and supports viral replication during early infection stages.

Overall, the balance of histone acetylation and methylation shapes the landscape of antiviral gene expression at mucosal and systemic sites. Disruptions to this balance by the virus can weaken the host’s frontline defenses, promoting viral persistence and contributing to the severity of COVID-19. Understanding these epigenetic influences offers promising avenues for therapeutic intervention, such as using epigenetic modulators to restore proper immune gene expression and enhance viral clearance.

### 5.3 Non-coding RNAs and mucosal immunity

miRNAs and long non-coding RNAs (lncRNAs, RNA transcripts longer than 200 nucleotides that do not encode proteins), previously regarded as insignificant by-products of transcriptional noise, have been identified as vital regulators of immune responses, particularly at mucosal surfaces where SARS-CoV-2 initiates infection (131). miRNAs and lncRNAs orchestrate fine-tuned regulation of immune genes, affecting the fragile equilibrium between protective immunity and pathological inflammation at the mucosal sites during COVID-19 (131). In the respiratory mucosa, miRNAs are crucial in modulating key cytokine signaling pathways (132) and shaping other antiviral mechanisms (133). For instance, specific miRNAs can inhibit the synthesis of pro-inflammatory cytokines like IL-6 and TNF-α, thus averting excessive inflammation that may harm sensitive lung tissue (134). On the other hand, some miRNAs boost antiviral defenses by making IFNs and other antiviral factors more active (135). Crucially, miRNAs also control autophagy, a crucial antiviral mechanism that breaks down pathogens and damaged organelles (136). miRNAs limit viral replication in the airway and preserve cellular homeostasis by regulating genes linked to autophagy at several levels, including chromatin remodeling, transcriptional control, and post-transcriptional modification (115).

LncRNAs regulate chromatin remodeling and transcriptional landscapes. It has been discovered that lncRNAs affect the expression of important antiviral genes during COVID-19, including those related to the IFN response (137). To limit viral spread and augment the mucosal antiviral state, for example, some lncRNAs can upregulate the transcription of ISGs (87). Furthermore, lncRNAs are important for controlling inflammasome pathways, which are essential for identifying viral infections and triggering inflammation (115). lncRNAs can either enhance protective inflammation required for viral clearance or, if dysregulated, shift protective immunity towards pathological hyperinflammatory state, seen in severe COVID-19 cases by altering the expression or activity of inflammasome components (115, 138).

Together, the interplay between miRNAs and lncRNAs forms a complex regulatory network that shapes mucosal immunity. Their precise balance determines whether the immune response effectively contains the virus with minimal tissue damage or escalates into harmful inflammation. Understanding these non-coding RNA-mediated mechanisms opens new avenues for therapeutic interventions, such as designing RNA-based drugs to restore immune balance and reinforce mucosal defenses against SARS-CoV-2.

### 5.4 Epitranscriptomics and m^6^A modifications at mucosal interfaces and local immune signaling

m6A methylation on host and viral RNAs modulates stability, translation, and immune recognition. Host methyltransferases (METTL3) and demethylases (ALKBH5) regulate these marks dynamically in response to infection (102, 103). Likewise, AMs show impaired antiviral gene expression due to m^6^A modifications of mRNA transcripts regulated by METTL3 and FTO enzymes, suggesting a role for RNA epitranscriptomics in dictating innate antiviral activity. SARS-CoV-2 alters their epigenetic programming to dampen their function. During SARS-CoV-2 infection, m^6^A dysregulation destabilizes IFN mRNAs or impairs ISG translation, weakening mucosal immunity (123, 139). m^6^A modifications are thus crucial for managing both host and viral RNA during SARS-CoV-2 infection (140). This occurs in mucous tissues like the respiratory tract, where the virus first enters and multiplies.

#### 5.4.1 m^6^A modulation of host antiviral transcript stability and translation

In functioning cells, m6A methylation affects the stability and translation of transcripts that encode antiviral and inflammatory mediators and the process is dynamic (141). Many genes that IFNs stimulate (ISGs) have m6A modifications such as IFIT1, ISG15, and OAS2. m6A methylation also shapes the expression of type I (IFN-α/β) and type III IFNs (IFN-λ) which are crucial for defending epithelial barriers against viruses (142). Unusual m6A methylation patterns in SARS-CoV-2 disrupt the balance needed to fight the virus effectively. It has been reported that reduced activity of demethylases like ALKBH5 during infection can lead to excessive methylation, making IFN-related mRNAs unstable and shutting down antiviral defenses (143). When METTL3, the main m6A methyltransferase, is absent, it weakens the translation of ISGs, making cells easy targets for the virus to replicate (123). The mucosal immune system in the respiratory epithelium relies on a quick and coordinated process to produce cytokines and chemokines (144) that help stop the virus locally before it spreads throughout the body. When m6A-related enzymes become unbalanced during infection, this process is disrupted. They may degrade antiviral mRNAs too soon or fail to produce them (141). This uneven activity causes the delayed IFN response seen in many severe COVID-19 cases, giving the virus a chance to multiply in the early stages of infection. Thus, SARS-CoV-2 co-opts m6A machinery to stabilize its genome and evade PRR-mediated detection, thus dampening type I/III IFN responses (140). In parallel, increased METTL3 activity in airway epithelia enhances viral RNA translation, contributing to immune evasion and viral persistence in mucosal tissues (145).

#### 5.4.2 Viral hijacking of m^6^A machinery

Scientists have noticed that SARS-CoV-2 takes advantage of the host’s RNA modification processes, especially m6A. These chemical changes are common throughout the viral genome and seem to help stabilize the virus’s RNA and improve its ability to produce proteins. Additionally, these modifications might aid the virus in evading the host’s immune defenses, which make early detection more challenging. At the same time, these modifications may help the virus slip past the host’s immune defenses, making early detection more difficult. m6A methylation also protects viral RNA from PRRs such as RIG-I and MDA5 (146). This protection lowers the activation of type I IFNs and other defenses of the innate immune system. Infections seem to modify the m^6^A regulatory network to create a pro-viral environment in cells. For instance, viral nsps and accessory proteins might affect how m6A writers or erasers function in the cell. Enhanced METTL3 and decreased FTO levels in airway epithelial cells have been reported during infections (147). This change could help the virus translate its RNA better while weakening immune-related transcripts in the host. This targeted adjustment of both viral and host transcripts might explain why some people experience severe immune suppression in mucosal areas even while facing extreme inflammation (cytokine storm) in the later stages of the illness (145).

#### 5.4.3 m^6^A in mucosal immunity: beyond epithelial cells

The role of m6A in epithelial cells has drawn significant attention during SARS-CoV-2 infection. However, its importance extends well beyond epithelial barriers, wherein, m^6^A modifications play a crucial role in regulating various immune cells at mucosal surfaces such as the alveolar macrophage and dendritic cell activation, innate lymphoid cells, mucosal B cell antibody responses, and mucosal T cell fate decisions, including exhaustion and regulatory phenotypes (148). These systemic and local impacts underscore the broader immuno-epigenetic programming occurring during SARS CoV-2 infection. These changes affect how immune cells detect, respond to, and handle viral threats in areas like the lung, nasopharynx, and gastrointestinal tract, which are the initial sites of SARS-CoV-2 infection.

SARS-CoV-2 reprograms mucosal immunity through site-specific epigenetic mechanisms, weakening epithelial defenses, blunting IFN responses, and reshaping immune cell function. Collectively, these studies emphasize the importance of epigenetic regulation at mucosal surfaces in determining susceptibility, immune response efficacy, and disease severity in SARS-CoV-2 infection. Integrating multi-layered epigenetic data from DNA methylation, histone modifications, non-coding RNAs to epitranscriptomic marks, will be crucial to unravel how host-virus interactions unfold within mucosal niches. Targeting these regulatory nodes with epigenetic therapeutics may offer novel avenues for restoring mucosal immunity and limiting viral pathogenesis.

## 6 Immune evasion strategies employed by SARS-CoV-2 at mucosal sites

**Figure 5:**
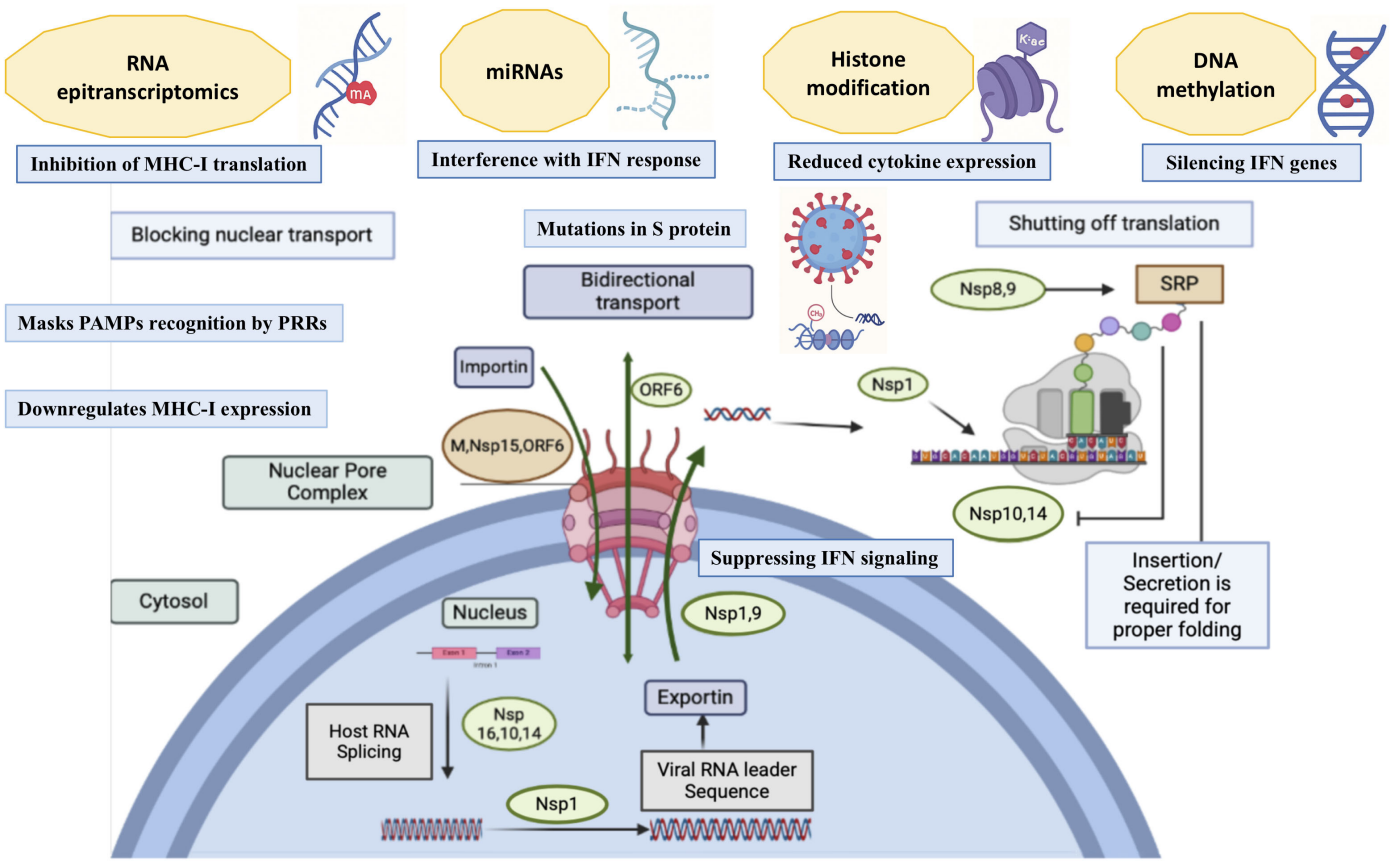
Immune evasion and immuno-epigenetic stratagems employed by SAR-CoV-2. Trafficking through the nuclear pore complex (NPC) is necessary for initiating an antiviral response. Infection-induced cellular transcription factors enter the nucleus via the NPC with the help of nuclear transport receptors (importins). Once in the nucleus, these factors bind to antiviral gene sequences to trigger their expression. The host spliceosome assembles at RNA splicing sites, excising introns to produce translationally competent mature transcripts after *de novo* transcription and capping of the developing messenger RNA (mRNA). These mRNA transcripts are then exported to the cytoplasm via the NPC, where the host ribosomes translate them for proper folding and cellular localization in response to the signal recognition particle’s (SRP) recognition of their signal peptide. Some SARS-CoV-2 proteins, including non-structural proteins (nsps), open reading frame 6 (ORF6) auxiliary protein, and the membrane structural protein (M), inhibit nuclear transport by interacting with host proteins like KPNA2, KPNA6, and others. These viral proteins also block RNA splicing, preferentially mitigating host RNA nuclear export while promoting viral RNA replication and interfere with ribosomal function, hinder protein trafficking, finally shutting down translation. Other immune elusion strategies include evasion of PAMP recognition by PRRs, inhibition of nuclear transport, spike protein mutations, downregulation of MHC Class I expression, and interference with interferon (IFN) signaling. Epigenetic mechanisms of immune evasion in SARS-CoV-2 and their impact are highlighted.

SARS-CoV-2, like other viruses, relies on host intricate cellular machinery for replication, necessitating strategies to elude the immune system and ensure successful infection. To achieve this, the virus manipulates and suppresses key host pathways, focusing on avoiding or mitigating the antiviral response. To subvert mucosal immunity, SARS-CoV-2 utilizes multiple strategies, from suppressing inflammatory mediators to interfering with MHC-I presentation and IFN signaling (**Figure 5**). The virus may also alter mucosal glycosylation patterns to escape antibody binding and sIgA neutralization (149). These tactics, combined with mutations in key viral proteins, and epigenetically driven immune evasion strategies enable the virus to persist, replicate, and spread efficiently, posing challenges for therapeutics and vaccine development. Understanding epigenetic warfare at the frontline - How SARS-CoV-2 tailors immune evasion in the mucosal microenvironment is crucial for designing effective countermeasures.

### 6.1 Blocking/shutting off host mRNA translation to proteins

The initial stage of SARS-CoV-2 infection involves the binding of the trimeric S glycoprotein to the host receptor ACE2. This process can proceed via a late entry pathway involving receptor-mediated endocytosis or an early entry pathway through direct membrane fusion. Cathepsin proteases may potentially be responsible for the twofold cleavage of the S protein during receptor-mediated endocytosis (150). For direct fusion, the S protein requires two proteolytic cleavages - the first cleavage is mediated by furin protease during viral maturation while the second cleavage, essential for fusion, is facilitated by TMPRSS2 at the cell surface. Following entry, the viral genome dissociates from the nucleoprotein complex, and the genomic viral ribonucleoprotein complex enters the cytoplasm wherein the genomic RNA is translated into proteins by host ribosomes (151).

The translation of viral mRNAs into polypeptides depends on the cellular ribosomes. To prioritize its own replication, SARS-CoV-2 selectively hijacks host translation machinery by targeting eukaryotic initiation factors (eIF), preferentially promoting viral mRNA translation while suppressing host defenses by impairing host immune-related protein synthesis (152). This strategy ensures suppression of host antiviral defense mechanisms by suppressing or halting host translational processes, which prevent the production of proteins involved in the host’s innate immune responses. Moreover, in the context of an acute viral infection, host cells turn off the translation system as a whole stress response to infection stress (153). This interference maintains a balance, allowing efficient viral replication without completely halting host cell machinery, and ensuring immune evasion.

Efficient synthesis of viral proteins such as nsp1, inhibit host mRNA translation by binding to the ribosome. Additionally, SARS-CoV-2 disrupts normal cellular responses, such as phosphorylation of eIF and degradation of host mRNA (154). While many mechanisms remain under study, it is clear that SARS-CoV-2 employs host shutoff to evade immune responses and maintain viral replication. Additionally, the virus’s exoribonuclease (nsp14) proofreads its genome for replication fidelity, thereby reducing replication errors and making it more genetically stable (155).

### 6.2 IFN signaling blockage

More than 60% of the total mRNA detected in an infected cell can be viral-derived, reflecting the efficiency with which the virus usurps the cell, as determined by single-cell RNA sequencing of SARS-CoV-2-infected cells. Significant amounts of transcription of viral genomic RNA and sgRNA, collectively with the nsp1-mediated inhibition of host mRNA, result in this outcome. SARS-CoV-2 effectively suppresses IFN signaling to avoid early immune detection through nsp1- and ORF6-mediated inhibition of host mRNA translation that prevents IFN-stimulated genes (ISG) expression. Proteins like nsp10 and nsp14 repress RNA splicing and host mRNA translation, ensuring that viral proteins are preferentially produced.

SARS-CoV-2 employs multiple strategies to suppress IFN responses, thereby evading early immune detection to establish infection. The S protein triggers the onset of SARS-CoV-2 infection and immune evasion by activating NF-kB signaling cascade through Toll-like receptor 2 (TLR-2) in dendritic cells, epithelial cells, monocytes, and macrophages contributing to inflammation but dampening antiviral signaling. The virus masks or reduces the recognition of pathogen-associated molecular patterns (PAMPs) by pattern recognition receptors (PRRs), thereby interfering with IFN production (156). Although the virus suppresses IFN responses early in infection, it eventually causes cell death, allowing phagocytes to detect and remove infected cells. Despite the immune suppression, SARS-CoV-2’s aggressive replication contributes to cellular invasion and widespread infection (157, 158).

### 6.3 Reducing MHC-I expression

SARS-CoV-2 downregulates MHC-I expression, impairing CTL activation and CTL-mediated clearing of infected cells. The virus achieves this by blocking the karyopherin complex-dependent nuclear translocation of Nod-like receptor family caspase recruitment domain-containing 5 (NLRC5), a critical MHC-I transactivator and by suppressing IFN-induced STAT1 signaling which lowers the expression of the genes for NLRC5 and IFN regulatory factor (IRF)-1, thereby targeting the critical MHC-I transcriptional regulators, STAT1-IRF-NLRC5. Downregulation of MHC-I molecules, particularly by ORF8, hampers cytotoxic T cell recognition of infected cells at the mucosal interface, allowing the virus to persist and replicate (159).

### 6.4 Preventing nuclear transport

A sizable structure known as the nuclear pore complex (NPC) connects the inner and outer membranes of the SARS-CoV-2 nuclear envelope to create an aqueous channel that controls nucleocytoplasmic trafficking. The nuclear transport receptors (importins and exportins) of the karyopherin protein family, which are in charge of transporting precisely tagged proteins between the nucleus and the cytoplasm, interact with the several protein subunits that make up the NPC, known as nucleoporins. SARS-CoV-2 disrupts the nuclear transport system, which is essential for host antiviral responses. By targeting the NPC and associated karyopherin transport proteins, the virus prevents or limits translocation of transcription factors critical for immune responses and impedes the export of host mRNA required for protein synthesis, impairing antiviral responses. ORF6 and ORF9b block nuclear translocation of transcription factors critical for IFN production. These manipulations provides SARS-CoV-2 with a selective advantage, enabling it to evade host defenses while continuing its replication (160).

### 6.5 Mutations in the S protein

Mutations in the S protein enhance immune escape by altering antibody recognition, without compromising ACE2 binding. Variants such as Alpha (B.1.1.7) that emerged in the UK in November 2020, Beta (B.1.351) first identified in South Africa, and Gamma (P.1) reported in Japan, harbor mutations like N501Y (161) and E484K (43) in the S protein’s RBD and NTD, reducing antibody neutralization and challenging vaccine efficacy. Omicron, the dominant strain of SARS-CoV-2 globally and has demonstrated a more rapid spread compared to earlier variants due to extensive mutations in the S protein, reducing antibody binding and vaccine effectiveness (162). These mutations enable the virus to evade immunity from prior infections or vaccinations.

### 6.6 Epigenetically-driven immune evasion in SARS-CoV-2 infection

SARS-CoV-2 has evolved sophisticated strategies to evade host immune surveillance, many of which are now recognized to operate through epigenetic mechanisms. Rather than relying solely on classical mechanisms such as antigenic drift or suppression of IFN pathways, SARS-CoV-2 actively manipulates the host’s epigenetic landscape to suppress antiviral responses, alter gene expression, and reprogram immune cell function. These virus-induced changes affect multiple levels of host transcriptional regulation ranging from chromatin accessibility and histone modifications to DNA methylation and non-coding (nc) RNA networks, and contribute substantially to immune escape and pathogenesis.

#### 6.6.1 Host transcriptional silencing via nsp1 and ORF proteins

One of the most direct epigenetic strategies employed by SARS-CoV-2 involves its nsps, particularly nsp1, which binds to the 40S ribosomal subunit and degrades host mRNAs, thereby halting host protein translation, including ISGs (163). Moreover, ORF6 impairs nucleocytoplasmic trafficking by interacting with the nuclear pore complex, blocking STAT1/STAT2 translocation and preventing the epigenetic activation of IFN-responsive promoters (45). Hypermethylation of ISG promoters, such as *IFNB1*, *ISG15*, and *MX1*, has been reported in infected airway epithelial cells, resulting in delayed type I and III IFN responses (87).

#### 6.6.2 Histone modulation to repress IFN pathways

SARS-CoV-2 may hijack host epigenetic modifiers, including HDACs and HMTs, to silence antiviral genes. Recent studies using ATAC-seq and ChIP-seq have demonstrated that SARS-CoV-2 infection leads to decreased chromatin accessibility at IFN-β, ISG15, and IRF7 loci, correlating with increased levels of repressive histone marks such as H3K27me3 and reduced activating marks (H3K4me3, H3K27ac) at antiviral IFN gene loci, which correlates with silencing of antiviral genes transcription and blunted type I and III IFN responses early in infection (85). SARS-CoV-2 has been shown to induce host epigenetic enzymes, such as EZH2 (the catalytic subunit of PRC2), which deposits a repressive mark, H3K27me3 on immune gene loci (ISGs and IL-6, TNF genes) in the airway epithelial cells and alveolar macrophages, directly contributing to the silencing of IFN responses (164). Reduced H3K9ac at PRDM1 (Blimp-1) has been reported to suppresses IgA-secreting plasma cell generation in Peyer’s patches and mucosal follicles (165).

#### 6.6.3 DNA methylation alterations in PRR and antigen presentation pathways and macrophage polarization

Host DNA methylation profiling in SARS-CoV-2-infected lung mucosal epithelial and immune cells revealed hypermethylation in promoter of PRR genes including TLR3, RIG-I and MDA5, potentially reducing their transcriptional responsiveness (86). For instance, DNA hypermethylation and histone deacetylation at TLR3 and RIG-I promoters have been observed in SARS-CoV-2-infected airway epithelial cells, reducing their expression and impairing viral RNA sensing (86, 166). This weakens the epithelial alarm system and facilitates viral immune evasion. Additionally, genes involved in antigen presentation (HLA-A, HLA-B and NLRC5) are epigenetically suppressed through increased promoter methylation, compromising MHC-I expression and CTL priming (167). SARS-CoV-2-induced hypermethylation of IRF5 and NOS2 genes in mucosal macrophages and dendritic cells have been reported to suppress M1-like pro-inflammatory programming, skewing macrophages toward regulatory or tolerogenic states, which benefit viral persistence (168).

#### 6.6.4 ncRNAs modulating immune gene networks

RNA-based regulators are essential for tuning the delicate balance between protective immunity and excessive inflammation at mucosal sites. SARS-CoV-2 also alters the expression of miRNAs and long non-coding RNAs (lncRNAs) that regulate antiviral defense. Notably, SARS-CoV-2 infection upregulates immunosuppressive miRNAs such as miR-146a, miR-21 and miR-155, which suppress IRAK1, TRAF6 and STAT1, thus dampening TLR signaling, NF-κB activation and IFN responses (169). Similarly, dysregulation of NEAT1 and MALAT1 lncRNAs expressed in epithelial and myeloid cells, contributes to inappropriate inflammasome activation and skewed cytokine production in the lungs, resulting in hyperinflammation or immunosuppression (99). miRNA networks have been found to regulate macrophage polarization (such as miR-125b that targets IRF4) and CD8^+^ T cell exhaustion (miR-31 that modulates PD-1 expression), influencing mucosal immune tolerance or dysfunction in COVID-19 (170, 171).

#### 6.6.5 RNA epitranscriptomic modifications and immune escape

RNA epitranscriptome acts as a rapid, reversible layer of regulation that is exploited by the virus to adapt to the local mucosal immune environment. Recent studies have identified that SARS-CoV-2 co-opts m^6^A RNA methylation to regulate the stability and translatability of viral and host mRNAs. Viral RNA is heavily modified with m^6^A, which helps it enhance translation efficiency of viral proteins and evade innate sensors like RIG-I. m^6^A writers such as METTL3 are hijacked to reduce recognition by RIG-I and modulate ISG expression (106, 172). Host ISG transcripts such as IFITM3, OASL and MX1 are modified by m^6^A, with altered methylation patterns affecting their translation and degradation during SARS-CoV-2 infection (87). In macrophages and dendritic cells, m^6^A affects the stability of transcripts encoding PRRs, co-stimulatory molecules (CD80, CD86) and cytokines (IL-1β, IFN-λ), thereby shaping the intensity and quality of mucosal immune responses (170). The RNA epitranscriptomic remodeling creates a post-transcriptional environment favorable for viral replication and immune evasion.

#### 6.6.6 Dysregulated epigenetic cues affecting adaptive immune programming at mucosal sites

The epigenetic landscape of mucosal-resident immune cells shapes adaptive responses to SARS-CoV-2. Tissue-resident T cells in the lungs of COVID-19 patients exhibit altered chromatin accessibility at genes encoding cytokines like IFN-γ and IL-2, along with increased exhaustion markers (PDCD1, LAG3), driven by repressive histone marks (173). Similarly, mucosal B cell class-switching to IgA in mucosal-associated lymphoid tissue (MALT) is regulated by methylation of AICDA gene, which encodes activation-induced cytidine deaminase (AID), and histone acetylation at immunoglobulin gene loci (71, 174). Dysregulation of these epigenetic cues may impair sIgA production, weakening mucosal humoral immunity. In addition, the downregulation of lnc RNA such as NEAT1 has been implicated in defective B cell activation and mucosal antibody production during infection (175). These findings highlight how SARS-CoV-2 may epigenetically disrupt protective mucosal humoral immunity.

#### 6.6.7 Epigenetic reprogramming facilitating viral persistence

SARS-CoV-2 manipulates host epigenetics to maintain a permissive transcriptional environment. For instance, nsp5 and nsp14 interfere with host chromatin remodelers, reducing histone acetylation at immune genes and promoting persistence (90). Viral infection has also been reported to result in suppression of chromatin accessibility at antiviral enhancers while preserving access to metabolic and ribosomal gene loci, reflecting a strategy to sustain viral replication without triggering full-scale immune activation (176).

#### 6.6.8 Epigenetic regulation of mucosal barrier function and immune cell activity in SARS-CoV-2

Respiratory epithelial cells form tight junctions critical for maintaining the mucosal barrier. During SARS-CoV-2 infection, epigenetic repression of tight junction proteins such as claudin-1 (CLDN1) and occludin (OCLN) has been observed, contributing to barrier disruption and viral dissemination (177). For instance, DNA hypermethylation at CLDN1 and OCLN promoters correlates with decreased expression in infected nasal epithelial cultures. Furthermore, SARS-CoV-2-induced histone deacetylation, particularly reduced H3K27ac at barrier gene loci, has been linked to increased epithelial permeability and impaired ciliary clearance (80). Such changes weaken frontline defenses and facilitate viral access to deeper tissues.

### 6.7 Epigenetic plasticity of SARS-CoV-2: distinct immune modulation at mucosal versus systemic sites

The immune evasion by SARS-CoV-2 is not merely a matter of viral stealth but rather a targeted, epigenetically regulated process. Epigenetic modifications at mucosal surfaces orchestrate the interplay between viral entry, innate immune alertness, and adaptive responses during SARS-CoV-2 infection. By manipulating host chromatin structure, ncRNA networks, and epigenetic enzymes, the virus enhances its replication potential and attenuates early antiviral responses, particularly in the respiratory tract, and thus, rewires the host transcriptional landscape to its advantage. Understanding these interactions underscores the potential of epigenetic therapeutic strategies or adjuvants aimed at restoring immune competence (such as mucosal IFN responses) or limit receptor expression to mitigate early viral spread, thereby offering insights into the long-term immunological consequences of COVID-19. However, viral immuno-epigenetic strategies might differ or be particularly potent in the mucosal environment compared to systemic responses due to tissue-specific potency of immuno-epigenetic strategies in the mucosal environment during SARS-CoV-2 infection.

The mucosal surfaces of the respiratory tract, the primary entry point and early battleground for SARS-CoV-2, are a focal point for the virus’s immuno-epigenetic strategies. Compared to systemic tissues, the mucosal environment exhibits a unique immune architecture dominated by epithelial-immune crosstalk, tissue-resident lymphocytes, and a tolerogenic milieu, which the virus exploits through targeted epigenetic reprogramming. Recent studies demonstrate that epigenetic suppression of type III interferon (IFN-λ) responses in airway epithelial cells is more pronounced than in peripheral immune cells, a process associated with increased H3K27me3 and promoter DNA methylation at IFNL1 and IFNL2 loci (178). This selective silencing impairs localized antiviral defenses without triggering strong systemic cytokinemia, allowing early viral propagation while avoiding early immune detection.

Moreover, nasal and bronchial epithelial cells display distinct chromatin accessibility profiles compared to peripheral blood mononuclear cells (PBMCs), following SARS-CoV-2 infection inducing chromatin remodeling at mucosa-specific regulatory elements (167). Mucosal macrophages and dendritic cells, already epigenetically tuned for tolerance and low inflammatory thresholds, undergo further repression of pro-inflammatory genes encoding TNF and IL-12 via increased H3K9me2 and recruitment of HDACs, enabling the virus to suppress early local innate immune responses (80). Importantly, ncRNAs such as miR-146a and miR-155, which are differentially expressed in the respiratory epithelium, target TLR and NF-κB signaling more robustly in mucosal settings, contributing to dampened antigen presentation and costimulatory signaling in dendritic cells (132).

In contrast, systemic immune responses typically display delayed but hyperinflammatory responses, a second wave of cytokine production that reflects systemic spillover, often exacerbated by a lack of early mucosal containment. The failure of early mucosal IFN responses, driven by epigenetic suppression, may thus underlie the progression from asymptomatic to severe disease (179). These findings suggest that SARS-CoV-2 has evolved epigenetic evasion strategies that are fine-tuned to the immune context of mucosal surfaces because of tolerogenic immune tone, specialized cytokine profile, and epigenetic plasticity of resident cells in the mucosal environment, prioritizing stealth over systemic disruption during early infection. Understanding this tissue-specific epigenetic plasticity may inform the development of mucosal-targeted antivirals and epigenetic adjuvants to bolster frontline defenses.

## 7 Epigenetic contributions to long COVID: sustained mucosal inflammation and immune dysregulation

The mucosal interface is a pivotal battleground in the pathogenesis and containment of SARS-CoV-2. The virus’s ability to establish infection, modulate local immunity, and evade early antiviral responses at mucosal sites underpins its transmissibility and clinical variability. Emerging evidence suggests that epigenetic dysregulation plays a critical role in the pathogenesis of long COVID, particularly in the persistence of mucosal inflammation, impaired immune homeostasis, and dysfunctional epithelial repair long after SARS-CoV-2 clearance (81). Several interconnected epigenetic mechanisms including DNA methylation, histone modifications, ncRNAs, and RNA epitranscriptomics contribute to these sequelae by shaping host immune memory, cellular exhaustion, and tissue remodeling at mucosal sites.

### 7.1 Persistent inflammatory epigenetic programming

Studies indicate that SARS-CoV-2 infection induces long-lasting epigenetic reprogramming in monocytes, AMs, and mucosal epithelial cells, favoring a pro-inflammatory phenotype. For instance, genomic and epigenomic profiling of COVID-19 survivors has revealed persistent open chromatin states at IL-1β, TNF, and CXCL10 loci, marked by sustained H3K4me3 and H3K27ac, supporting continued cytokine expression even in the absence of active infection (180). This may underpin chronic symptoms such as fatigue, dyspnea, and cough.

### 7.2 Trained immunity and myeloid hyperresponsiveness

c. Such reprogramming is supported by elevated chromatin accessibility at key pro-inflammatory loci and persistent STAT1 and NF-κB activation (181). While protective initially, this maladaptive training may promote low-grade chronic inflammation and exaggerated responses to commensals or allergens, contributing to respiratory and gastrointestinal symptoms.

### 7.3 T cell exhaustion and epigenetic scarring

Sustained DNA methylation at effector gene loci such as IFN-γ, GZMB and enhancer repression through H3K9me3 deposition have been reported in mucosal CD8^+^ and CD4^+^ T cells from long COVID patients (182). This epigenetic scarring resembles exhausted T cell profiles seen in chronic viral infections and may impair adaptive responses and barrier surveillance.

### 7.4 Silencing of epithelial repair pathways

In bronchial and intestinal epithelial cells, SARS-CoV-2 has been shown to modulate DNA methylation and histone acetylation of genes regulating tight junctions such as CLDN1, OCLN, mucins, and anti-inflammatory mediators like IL-10 (80). Prolonged silencing of these genes can compromise epithelial regeneration, perpetuating barrier disruption and microbial translocation.

### 7.5 ncRNA and RNA modification-mediated dysregulation

Persistent alteration in miRNAs such as miR-21, miR-146a and lncRNAs (NEAT1, MALAT1) modulates the JAK/STAT and TLR signaling pathways, influencing inflammatory gene expression and immune cell function post-infection (86). Additionally, m^6^A RNA methylation of key transcripts may control their stability and translation, skewing mucosal immune responses (183).

Collectively, these findings underscore that epigenetic rewiring at mucosal surfaces is not merely a byproduct of infection but a central mechanism in the persistence of inflammation and immune dysfunction in long COVID. Understanding these processes offers a basis for biomarker discovery and opens avenues for epigenetic therapies aimed at restoring mucosal immune balance.

## 8 Key conceptual shifts or overarching principles emerging from immuno-epigenetics in SARS-CoV-2 infections

The study of immuno-epigenetic interactions in SARS-CoV-2 infection has catalyzed several paradigm shifts in our understanding of viral pathogenesis, host defense, and long-term immune remodeling. Unlike classical models that focused predominantly on viral protein-host receptor interactions and downstream cytokine cascades, the COVID-19 pandemic has illuminated the central role of epigenetic regulation in orchestrating both innate and adaptive immune responses, with critical implications for immune evasion, disease heterogeneity, and post-infectious sequelae.

### 8.1 The immune response is epigenetically tuned, not just triggered

Traditional views conceptualized immune activation as a binary response to viral antigens. However, SARS-CoV-2 has demonstrated how the timing, magnitude, and tissue specificity of immune responses are epigenetically programmed. Chromatin accessibility, DNA methylation, and histone modifications dynamically influence the expression of PRRs, type I/III IFNs, and antiviral effectors, shaping outcomes ranging from asymptomatic infection to severe COVID-19 (184).

### 8.2 Viral epigenetic reprogramming extends beyond acute infection

SARS-CoV-2 infection induces persistent epigenetic changes in mucosal epithelial and immune cells, extending well into convalescence. This has shifted focus from short-lived transcriptional responses to long-term epigenetic memory, including trained immunity in myeloid cells and exhaustion signatures in T cells. These durable alterations provide a mechanistic basis for long COVID, especially in individuals experiencing sustained inflammation or immune dysregulation (185).

### 8.3 Mucosal immunity is an epigenetically distinct compartment

Emerging research has revealed that mucosal tissues such as the respiratory and gastrointestinal tracts harbor unique epigenetic landscapes, which modulate their immunological responsiveness to SARS-CoV-2. IFN-λ genes, mucin production, and epithelial barrier function are under the control of site-specific histone modifications and chromatin remodeling complexes, highlighting the need for mucosa-specific therapeutic strategies (6).

### 8.4 Immune evasion involves epigenetic silencing mechanisms

Beyond classic immune evasion strategies, SARS-CoV-2 encodes proteins that directly interfere with host epigenetic machinery, including HDACs, chromatin remodelers, and RNA modification enzymes, to suppress antigen presentation and IFN signaling (90). This expands our understanding of how viruses subvert host immunity at the transcriptional and epigenomic level, rather than relying solely on structural protein variation.

### 8.5 Therapeutic modulation of epigenetic pathways is a viable antiviral strategy

The realization that key immune genes are epigenetically regulated has spurred interest in targeting epigenetic modifiers such as HDAC inhibitors, bromodomain inhibitors, methyltransferase blockers, to restore antiviral immunity and resolve chronic inflammation. This marks a conceptual shift from antiviral drugs targeting viral enzymes alone to host-directed therapies that reprogram the immune system’s epigenetic state (143).

These conceptual advances redefine the immune response to SARS-CoV-2 not as a linear cascade, but as a multilayered, plastic, and dynamically regulated system with epigenetic mechanisms at its core. This shift compels the integration of epigenomic profiling into infectious disease research and opens new avenues for diagnostic biomarkers, precision immunotherapies, and long COVID management strategies.

## 9 Influence of SARS-CoV-2 virulent proteins on the host’s epigenetic landscape

SARS-CoV-2 possesses the largest genome of any known coronavirus, encoding numerous structural and non-structural proteins that play crucial roles in evading pathogen detection and modulating various antiviral responses. A recent study by Gordon et al. has mapped viral proteins to their human host partners, identifying approximately 332 human proteins that interact with viral components, including eight associated with epigenetic machinery (186). Similarly, Albarrán et al. analyzed transcriptomes from SARS-CoV-2-infected cell lines, identifying pathways enriched with transcription factors relevant to the infection and more than 60 proteins linked to epigenetic processes that may offer therapeutic potential (187).

One notable interaction involves nsp5, a protease encoded by SARS-CoV-2, which interacts with HDAC2, thereby impairing IFN production and inflammatory responses. HDACs are crucial for regulating gene expression by functioning as transcriptional repressors through the removal of histone acetylation, with HDAC2 specifically promoting various interferon-stimulated genes (ISGs) via BRD4 to mount an effective antiviral response. It is hypothesized that Nsp2 may cleave the nuclear localization signal of HDAC2, preventing its nuclear translocation and inhibiting several antiviral mechanisms. Additionally, nsp5 is predicted to cleave the nuclear localization signal of tRNA methyltransferase 1 (TRMT1), which disrupts its transport to the nucleus, resulting in its localization in mitochondria (187).

Furthermore, recent research indicates that the C-terminal region of the viral envelope protein E mimics the N-terminal tail of histone H3, competitively binding to bromodomain proteins BRD2/4 instead of histone H3, thereby disrupting downstream transcriptional activities (79). BRD2/4 typically recognize acetylated histones to activate transcription.

SARS-CoV-2 also encodes viroporins like ORF8a and ORF3a, which are hydrophobic proteins that activate the NLRP3 inflammasome, leading to a cytokine storm associated with tissue inflammation and respiratory complications. Another viral protein, ORF3b, is known to regulate type I interferon production. The ORF3b protein in SARS-CoV-2 is notably shorter, consisting of 22 amino acids due to a premature stop codon compared to its SARS-CoV counterpart, which is 154 amino acids long. This shorter variant in SARS-CoV-2 is believed to contribute to its increased virulence. Konno et al. demonstrated that introducing a premature stop codon into the SARS-CoV-ORF3b sequence significantly enhanced its ability to inhibit the type I interferon response compared to the wild-type version. It is proposed that ORF3b can effectively suppress various host defense mechanisms early in the infection by inhibiting IFN production through epigenetic mechanisms, prior to the activation of B and T cells (188).

Additionally, proteins such as ORF10 and Nsp13 are reported to interact with the human Cullin-RING E3 ubiquitin ligase complex and ubiquitin-specific peptidase 13 (USP13), respectively. These interactions suggest that SARS-CoV-2 may exploit the protein degradation pathways to induce the proteasomal degradation of host restriction enzymes, thereby facilitating viral replication. nsp13 is also believed to influence IFN signaling and NF-κB-mediated inflammatory responses through interactions with TLE, TBK1, and CENPF. Furthermore, ORF8 and Nsp8 are predicted to interact with DNMT1 and MEPCE, respectively. The interaction between ORF8 and DNMT1 is particularly noteworthy due to its potential long-term effects post-recovery. DNMT1 is responsible for maintaining physiological DNA methylation patterns and is involved in the replication process during mitosis to ensure the preservation of epigenetic patterns in daughter cells. Disruptions in DNMT1 activity during COVID-19 could lead to conditions such as atherosclerosis, liver dysfunction, and diabetes. Similarly, MEPCE is a critical regulator of RNA polymerase II-mediated transcription elongation, and its dysregulation may adversely affect the transcriptional regulation of the virus (186).

### 9.1 Epi-transcriptomic mechanistic insights into SARS-CoV-2 disruption of host defense mechanisms

Histone mimicry involves the structural and functional resemblance of viral proteins to host histones. This mimicry allows the virus to exploit host cellular machinery, disrupting normal regulatory processes such as transcription and chromatin remodeling (176). Certain SARS-CoV-2 proteins, particularly the E protein and nsps (such as nsp5), have regions that structurally mimic the tails of histones. These regions can interact with bromodomain-containing proteins (BRDs), which are essential for recognizing acetylated histones and facilitating transcriptional activation. By mimicking histone tails, SARS-CoV-2 proteins can competitively bind to bromodomains (e.g., BRD2 and BRD4) that are critical for recognizing acetylated lysine’s on histones, facilitating the recruitment of transcriptional co-activators and RNA polymerase II. The viral proteins’ mimicry of histone tails allows them to bind to these bromodomains, effectively blocking their interaction with true histones and hindering the transcriptional machinery’s access to DNA, necessary for the expression of host genes, including those involved in immune responses (189). Binding of viral proteins to host transcription factors and chromatin remodeling complexes disrupts the activation of interferon-stimulated genes (ISGs). This impairs the host’s ability to mount an effective antiviral response, making it easier for the virus to replicate and spread. By affecting histone acetylation and methylation patterns, SARS-CoV-2 can change the chromatin structure of infected cells, leading to transcriptional silencing of genes critical for immune surveillance and response.

The disruption of normal transcriptional regulation can lead to a dysregulated immune response, contributing to cytokine storms—a severe overproduction of pro-inflammatory cytokines (**Figure 4**). This phenomenon is observed in severe COVID-19 cases and results in extensive tissue damage and respiratory complications. The virus’s ability to inhibit ISGs through histone mimicry allows it to evade the innate immune response, prolonging the infection and increasing the risk of severe outcomes.

### 9.2 Long-term effects of histone mimicry on host cells

The effects of histone mimicry are not limited to immediate viral replication. Prolonged interaction with viral proteins can lead to lasting epigenetic changes in host cells, potentially affecting gene expression patterns even after the virus has been cleared. This may contribute to long-term complications associated with COVID-19 (80). Dysregulation of histone modification processes can lead to chronic conditions, including cardiovascular diseases, diabetes, and other metabolic disorders, which are observed more frequently in COVID-19 survivors. Understanding the mechanisms of histone mimicry by SARS-CoV-2 may open avenues for novel therapeutic strategies. Inhibitors that disrupt the interaction between viral proteins and host epigenetic machinery could potentially restore normal immune function and improve outcomes in infected patients (190). Agents that modify histone acetylation or methylation patterns could be explored to counteract the effects of viral hijacking of host transcriptional regulation. Such treatments could help re-establish normal immune responses and reduce the severity of inflammation.

By mimicking histones, SARS-CoV-2 proteins can inhibit the expression of type I and type III IFNs, which are crucial for orchestrating the immune response against viral infections. The suppression of IFN signaling leads to decreased activation of ISGs that would normally enhance the antiviral state of the cell. Histone mimicry may also impact the expression of various pro-inflammatory cytokines and chemokines (191). Disruption in the expression of these molecules can result in an inadequate immune response, allowing the virus to establish a stronger foothold in the host.

The interactions between viral proteins and host chromatin-remodeling factors can lead to long-lasting changes in the epigenetic landscape of infected cells. For instance, aberrant histone modifications could silence genes that are critical for immune surveillance long after the virus is cleared, potentially contributing to chronic conditions like post-viral syndrome or long COVID (80). Research suggests that the dysregulation of histone modification enzymes, such as DNMT1, during SARS-CoV-2 infection can lead to an increased risk of comorbidities (192). These epigenetic changes have been linked to increased risks of cardiovascular diseases, atherosclerosis, metabolic disorders such as diabetes, and liver dysfunction in COVID-19 survivors.

Viroporin proteins, such as ORF3a and ORF8a, not only disrupt cellular ionic balance but also play a role in activating the NLRP3 inflammasome (193). This activation leads to a cascade of inflammatory cytokines, contributing to the hyperinflammatory state seen in severe COVID-19 cases. The mimicry of histone proteins may further exacerbate this by interfering with the normal resolution of inflammation (191).

SARS-CoV-2 utilizes histone mimicry as a sophisticated mechanism to manipulate host cellular processes, particularly in modulating immune responses and altering gene expression. This strategy not only facilitates viral replication but also contributes to the pathogenesis of severe COVID-19 outcomes and long-term health issues. Continued exploration of these interactions will be crucial for developing effective therapeutic strategies and understanding the broader implications of viral infections on host epigenetics. Understanding the mechanism of histone mimicry provides potential therapeutic avenues. Small molecules that inhibit the interaction between viral proteins and host bromodomain proteins could restore normal transcriptional regulation and enhance the antiviral response.

### 9.3 Interplay of epigenetic modifiers with other viral proteins

Nsp5’s interaction with HDAC2 inhibits the deacetylation of histones, which normally regulates the expression of antiviral genes. The blockage of HDAC2 by Nsp5 not only impairs interferon production but may also lead to an accumulation of acetylated histones, creating a feedback loop that further disrupts gene expression. ORF3b’s inhibition of type I interferons, coupled with its shorter variant in SARS-CoV-2, further enhances the virus’s ability to evade immune detection (194). This protein may also utilize histone mimicry to block the action of transcription factors that would normally promote antiviral responses.

Agents that modulate histone acetylation and methylation, such as HDAC inhibitors, may be repurposed to counteract the effects of SARS-CoV-2. These therapies could help reinstate proper immune signaling pathways and mitigate the long-term effects of infection. Further research is needed to explore the specific interactions between viral proteins and host epigenetic regulators. Identifying key viral-host interactions can inform the development of targeted therapies that could prevent COVID-19.

The virus’s interaction with DNMT1 and histone modification enzymes may create an “epigenetic memory,” perpetuating the dysregulated expression of immune-related genes. This may explain prolonged symptoms and susceptibility to reinfections.

## 10 Potential therapeutic implications with drugs targeting the epigenetic factors

Epigenetic therapies offer a promising avenue for combating SARS-CoV-2 by targeting the host’s epigenetic machinery rather than directly attacking the virus. From the study of tissue-specific epigenomes, it is evident that the disease has a substantial impact on these profiles. For instance, lung cells with cancer exhibit very different epigenetic traits than lung cells impacted by COVID-19 (195). Comprehending these distinctions is crucial for formulating accurate treatment plans.

Whether nsps or other proteins encoded by SARS-CoV-2 ORF6, ORF7, and ORF8 interact with epigenetic factors is an important topic of research. Investigating these relationships may provide important information for developing COVID-19 treatments based on epigenetics as displayed in **Figure 4**. Some of the drugs targeting the epigenome in SARS-CoV2 are represented in **Table 2**. These therapies may improve immune responses, control gene activity and prevent the virus from proliferating or persisting in the host.

**Table 2:** Drugs targeting epigenetic engines in SARS-CoV-2.

Epigenetic drugs	Epigenetic modulatory targets	References
Vitamin D	Inhibits ACE2 and furin	(260, 261)
Quercetin	Inhibiting ACE2 and furin	(262, 263)
Curcumin	Activates DNMTs by silencing ACE2 and interferon genes	(264, 265)
Hydroxyquinolines	Activate DNMTs by silencing ACE2 and interferon genes	(266)
Sulforaphane	Inhibits HDAC	(267)
Thymoquinone	Inhibits HDAC	(268)
β-sitosterol	Repress DNMTs, IL-6, IL-1β, TNF-α	(269)
Lupeol	Repress DNMTs, IL-6, IL-1β, TNF-α	(270)
Procyanidin B2	Inhibits DNMTs	(271)
Suberanilohydroxamic acid (SAHA) or Vorinostat along with antiviral drug	Inhibits HDAC	(272)
Azacytidine	Inhibits DNMTs	(273)
Anacardic acid	Inhibits HAT1	(274)
Valproic acid	Inhibits HDAC1/2	(275)
5-aza-2′-deoxycytidine (5-azadC) and/or decitabine	DNMT inhibitor(s) suppressing inflammation and IFN response by inhibition of DNA methylation in macrophages	(274, 276)
Vitamin C	Attenuating the activation of NF-κB	(277, 278)
Vitamin B	Gene silencing by histone deacetylation and DNA methylation	(277, 279)
Decitabine	Inhibits DNMT, inhibits DNA methylation in macrophages suppressing IFN response and inflammation	(274, 276)
Chaetocin	Inhibits HKMT	(274, 280)
3-deazaneplanocin-A (DZNep)	Inhibits HKMT	(274, 281)

ACE, Angiotensin converting enzyme; DNMT, DNA methyl transferase inhibitor; HDAC, Histone deacetylase; HAT, Histone acetyl transferase; HKMT, Histone Lysine methyl transferase; IFN, Interferon; IL, Interleukin.

### 10.1 Targeting the epigenetic-like mechanisms utilized by SARS-CoV-2 offers novel therapeutic opportunities

Understanding SARS-CoV-2’s RNA synthesis has been critical in developing antiviral drugs. Remdesivir, for example, targets RdRp (nsp12), preventing the virus from copying its RNA (196). Molnupiravir introduces mutations into the viral genome, leading to non-functional viruses (197, 198), while Paxlovid (Nirmatrelvir + Ritonavir) inhibits Mpro (nsp5, the main protease), blocking viral protein processing (199). These treatments, along with vaccines, have played a crucial role in reducing the severity and spread of COVID-19. The virus’s ability to efficiently replicate and evade immune defenses underscores the need for continued research to develop more effective antiviral strategies.

An array of epigenetic-like mechanisms utilized by SARS-CoV-2 for its replication may be targeted by therapeutic drugs. These include m6A inhibitors that inhibit host methyltransferases involved in m6A modification and may thereby reduce viral RNA stability and replication (200). RNA G-Quadruplex (RG4) stabilizers have been reported to attenuate SARS-CoV-2 infection in pseudovirus cell systems and mouse models (201). Hence, compounds that disrupt G-quadruplex structures in the viral genome may impair its replication machinery. Host-driven RNA editing can be enhanced by RNA editing modulators that may introduce lethal mutations into the viral genome, reducing its infectivity (202). nsp5, a viral protease, interacts with and impairs HDAC2 function, preventing its nuclear localization. HDAC2 is crucial for regulating ISG expression and the interferon response (203). Inhibition of HDAC2 by nsp5 dampens antiviral gene activation, enhancing viral replication. RNA Pol II dysregulation via methylphosphate capping enzyme (MEPCE) has been found to attenuate host’s antiviral response. The interaction between Nsp8 and MEPCE disrupts transcription elongation mediated by RNA Pol II, impairing the expression of antiviral genes (187). Viroporins such as ORF3a and ORF8a activate the NLRP3 inflammasome, triggering a cytokine storm (204). This hyperinflammatory state is exacerbated by epigenetic silencing of genes that resolve inflammation. The ORF3b protein in SARS-CoV-2 enhances its ability to inhibit interferon responses, silencing ISG expression through epigenetic mechanisms (205). This suppression contributes to immune evasion and viral persistence.

### 10.2 Targeting the epigenetic changes induced by SARS-CoV-2 offers promising therapeutic strategies

Epigenetic modifications may be harnessed as potential therapeutic targets to combat SARS-CoV-2 infection. For instance, bromodomain inhibitors targeting BRD2/4 have been reported to prevent viral proteins from disrupting host transcription, thereby restoring normal immune responses (203, 206). Further, epigenetic enzyme modulators such as HDAC inhibitors may counteract the effects of nsp5, reactivating silenced antiviral genes (207). Likewise, DNMT1 inhibitors may mitigate aberrant DNA methylation patterns induced during infection (86). Further, epi-drugs targeting NLRP3 inflammasome activation may reduce cytokine storm and associated tissue damage (208). The potential of epigenetic-based therapies in managing COVID-19 underscores the growing need for further research into their efficacy and safety in combating SARS-CoV-2 infection.

## 11 Limitations of current studies in the immuno-epigenetics of SARS-CoV-2

Despite rapid advances, several limitations constrain current understanding of immuno-epigenetic regulation in SARS-CoV-2 infection. First, most studies rely on bulk tissue or peripheral blood analyses, which overlook mucosal compartmentalization and cell-type-specific epigenetic changes critical at viral entry sites like the respiratory epithelium (113). Single-cell epigenomic mapping remains limited, especially for key mucosal immune subsets such as resident macrophages, ILCs and tissue T cells.

Second, temporal resolution is lacking in many studies, making it difficult to distinguish cause from consequence in epigenetic remodeling during acute versus convalescent phases (6). The dynamic interplay between viral components and host chromatin regulators such as METTL3, DNMTs, HDACs is often inferred indirectly and not validated functionally in *in vivo* systems.

Moreover, most mechanistic insights are extrapolated from *in vitro* or animal models, which may not fully recapitulate human mucosal epigenetic responses (87). There is also a paucity of studies integrating epigenetic data with clinical outcomes such as long COVID or vaccine responsiveness.

Finally, while associations between epigenetic marks and gene expression are increasingly reported, direct causality remains underexplored, and few therapeutic studies target these pathways specifically in SARS-CoV-2 infection (86, 108).

## 12 Conclusion and future perspectives

SARS-CoV-2 has highlighted the pivotal role of immuno-epigenetic regulation in determining the outcome of viral infections, especially at mucosal surfaces, the critical first line of defense. Epigenetic reprogramming of immune and epithelial cells at these sites shapes antiviral responses, barrier function, and inflammation resolution, with lasting consequences for disease severity and recovery. Persistent alterations may contribute to long COVID by sustaining immune dysregulation and tissue dysfunction.

The COVID-19 pandemic continues to pose global health and economic challenges despite advancements in vaccines and therapeutics. While epitope-based vaccines and antiviral drugs show promise, incomplete understanding of viral protein functions hinders progress. A major contributor to SARS-CoV-2 pathogenicity is its ability to manipulate host epigenetic machinery through histone modifications, DNA methylation, and non-coding RNAs to suppress immune responses and expression of antiviral genes and enhance replication. These alterations not only drive acute infection but also contribute to chronic inflammation, metabolic dysfunction and immune dysregulation.

Emerging strategies such as combining antiviral agents with epigenetic modulators such as DNMT or HDAC inhibitors, offer potential by minimizing drug resistance, reducing viral replication and limiting toxicity but require further validation. Understanding virus-host epigenetic interactions is critical for developing precision-based targeted epigenetic therapies. Future research should focus on elucidating these mechanisms to improve treatment efficacy, boost immunity, and mitigate severe outcomes. Epigenetic-targeted therapies may hold promise not only for COVID-19 but for emerging viral threats.

Looking forward, future research must move beyond associative studies to mechanistic, high-resolution, and longitudinal investigations. Key priorities include spatial single-cell epigenomics of mucosal tissues, functional dissection of histone and methylation marks in epithelial and innate immune cells, and the identification of stable epigenetic biomarkers predictive of long COVID trajectories. CRISPR-based epigenome editing and mucosa-targeted delivery of epigenetic modulators offer promising experimental strategies to test causality and therapeutic efficacy.

Finally, integrating multi-omic datasets into systems-level models will enable a holistic understanding of how epigenetic landscapes govern immune memory, tolerance, and repair at mucosal barriers. These efforts will not only deepen insight into SARS-CoV-2 pathogenesis but may establish generalizable principles for combating respiratory viruses through epigenetically-informed interventions.
